# An evaluation of the Syrian pregnant women’s prenatal care satisfaction: a cross-sectional study*

**DOI:** 10.1590/1980-220X-REEUSP-2025-0002en

**Published:** 2025-05-16

**Authors:** Sibel İçke, Sema Çifçi

**Affiliations:** 1 Mardin Artuklu University, Faculty of Health Sciences, Midwifery Department, Artuklu, Mardin, Türkiye.; 2 Mardin Artuklu University, Faculty of Health Sciences, Nursing Department, Artuklu Mardin, Türkiye.

**Keywords:** Transients and Migrants, Pregnant People, Prenatal Care, Personal Satisfaction

## Abstract

**Objective:**

This study aims to examine the satisfaction levels of Syrian migrant pregnant women living in Mardin with prenatal care services and the factors influencing their satisfaction.

**Method:**

This is a cross-sectional study. The population of the study consisted of Syrian pregnant women who applied to Mardin Training and Research Hospital between August 15 and September 16, 2023. A total of 146 Syrian pregnant women who met the inclusion criteria participated in the study. The sociodemographic information form and the Prenatal Care Satisfaction Scale were used as data collection tools.

**Results:**

The rate of those who received prenatal care from a midwife/nurse is 80.1% and those who received less than 4 prenatal care was 89.7%. The most common reason for not receiving adequate prenatal care was lack of information with a rate of 39.7%. The mean score of the PCSS was 73.39 ± 14.78.

**Conclusion:**

The study findings indicate that lack of information is one of the major barriers to healthcare access for migrant pregnant women. In addition, receiving prenatal care services from midwives/nurses affected satisfaction with prenatal care.

## INTRODUCTION

Prenatal care (PC) refers to the healthcare services provided by health professionals to pregnant women throughout their pregnancy to monitor and maintain the optimal health of both the mother and the infant^(1,2)^. High-quality PC is crucial as it incorporates health-promoting interventions for the mother and fetus, identifies potential adverse conditions at an early stage, and facilitates timely interventions^(2)^. Although global maternal mortality rates decreased by approximately 34% between 2000 and 2020, these rates remain significantly high in low- and middle-income countries, accounting for 95% of all maternal deaths^(3)^.

The civil war that began in Syria in 2011 led to an economic downturn^(4,5)^ and triggered widespread migration. Turkey, undoubtedly, has been the country most affected by the migration of Syrians^(5,6)^. As of the year 2024, the number of Syrians under temporary protection in Turkey is approximately 3.1 million, with nearly 1.5 million of them being women. Among these women, approximately 725,000 are aged between 15 and 49 years^(7)^.

One of the biggest problems Syrian migrants encounter various challenges while relocating to other countries is health-related issues^(8)^. The lack of access to PC is one of these challenges.

Being a migrant is one of the factors influencing access to PC. Migrant women benefit PC services less frequently due to barriers such as language, education, and transportation^(9,10)^. According to the 2018 Turkey Demographic and Health Survey (TDHS) data, 11% of Syrian migrants don not receive any PC, and 33.8% receive insufficient care, being significantly lower compared to native pregnant women^(11)^. In a study conducted with Syrian refugee women, the rate of those who did not receive PC was 41.3%, whereas it was 7.7% among Turkish citizens^(12)^. The World Health Organization (WHO) recommends receiving prenatal care (PC) at least eight times during pregnancy. However, global prenatal care coverage is still not at a sufficient level. According to the data from the World Health Organization and World Health Statistics in 2016, only 64% of women worldwide received PC four or more times during their pregnancies^(13)^.

Satisfaction is crucial in all areas of healthcare, including PC^(9)^. Studies reveal that migrant women report lower satisfaction with healthcare services due to delayed PC, insufficient information, and communication challenges^(14,15,16)^. Dissatisfaction with PC services may lead to difficulties in adapting to healthcare systems in the country migrated. The present study aims to examine the satisfaction levels of Syrian migrant pregnant women living in Mardin with PC services and the factors influencing their satisfaction.

Research Questions:

1. What are the satisfaction levels of Syrian pregnant women with PC services?
2. What factors influence the satisfaction levels of Syrian pregnant women with PC services?

## METHOD

### Research Type

This is a cross-sectional study.

### Population and Research Sample

The study population consisted of Syrian pregnant women who visited Mardin Training and Research Hospital between August 15, 2023 and September 16, 2023. Because the study was conducted exclusively with Syrian migrant pregnant women admitted to the hospital, the participants were selected using the purposeful sampling method. A total of 146 Syrian pregnant women who met the inclusion criteria participated in the study. In order to evaluate the statistical power of the study, posthoc power analysis for one-sample t-test was performed using G*Power 3.1 software. In the analysis, a moderate effect size (d = 0.5), statistical significance level (α = 0.05) and total sample size (n = 146) were used. The power obtained was calculated as (1-β) = 0.9999. This result shows that the study has a fairly high statistical power to detect a moderate effect size.

### Inclusion Criteria

1. Being Syrian,
2. Having a gestational period between 36 and 40 weeks,
3. Having no language barriers.

### Exclusion Criteria

1. Not being Syrian,
2. Having a gestational period outside the 36–40-week range,
3. Having language barrier.

### Data Collection Tools

The study used a questionnaire comprising two sections. The first section collected socio-demographic information, while the second section included the Prenatal Care Satisfaction Scale (PCSS), whose Turkish validity and reliability were assessed by Aslantekin Özçoban et al.^(17)^ and published in 2020.

***- Individual Identification Form (IIF):*** This form, developed by the researchers based on the literature, included socio-demographic and obstetric information and consisted of 36 questions.

***- Prenatal Care Satisfaction Scale (PCSS):*** The scale was originally developed by Raube et al.^(18)^ in 1998 to measure pregnant women’s satisfaction with PC^(17,18)^. It was adapted into Turkish and validated for reliability by Aslantekin Özçoban et al.^(17)^ in 2020. It is a self-reported Likert-type scale comprising 22 items. Based on the literature, it assesses five subscales: the art of care, technical quality, physical environment, accessibility, and suitability.

*Art of Care Subscale:* This subscale includes the perceived value of the services received in relation to the cost/premium paid, the ability of staff to provide comfort and a sense of security, the attentiveness of doctors, midwives, and nurses, the respectful behavior of healthcare professionals, and the empathy and understanding shown by reception staff during emotional states such as anxiety, excitement, or anger. It also evaluates the respectfulness of reception staff and the interest and approach exhibited by the welcoming/registration personnel.

*Technical Quality Subscale:* This includes the adequacy of explanations provided by midwives, nurses, or doctors regarding procedures, the competence of doctors, midwives, and nurses in delivering care and managing childbirth, the attentiveness of medical examinations, and the adequacy of medical equipment and supplies.

*Accessibility Subscale:* This subscale assesses transportation conditions, waiting period for examination in an appointment, the duration between the initial appointment date and the scheduled date for the next follow-up, and the suitability of opening hours (early or late).

*Physical Environment Subscale:* It evaluates the cleanliness, comfort, and convenience of the facilities, the condition of examination areas, and the adequacy of waiting rooms.

*Suitability Subscale:* It includes the time allocated by staff providing nutritional advice during pregnancy, the professional competence of doctors, midwives, and nurses, and the feasibility of the advice provided by healthcare professionals throughout the pregnancy to protect the health of both the mother and the infant.

The Cronbach’s alpha values for the subscales range between 0.7 and 0.9, while it is 0.95 for the overall scale. There is no cut-off point in the scale. The lowest score was 22, while the highest was 110. Responses on the scale are rated as follows: poor (1), fair (2), good (3), very good (4), and excellent (5). As the score on the scale increases, satisfaction is considered high, and as the score decreases, satisfaction is considered low (18).

### Ethical Consideration

Prior to the study, approval was obtained from the Mardin Artuklu University Non-Interventional Clinical Research Ethics Committee with decision number 2022-9, dated 01/08/2022. Financial support for the study was provided through the project MAÜ.BAP.22.SBF.026, aimed to carry out the research, by the Mardin Artuklu University Scientific Research Projects Coordination.

### Data Collection

The study data were collected using a face-to-face survey method at the obstetrics and gynecology outpatient clinics, NST (non-stress test) clinic, and prenatal classes. The questionnaires were conducted in Turkish face-to-face with pregnant women who agreed to participate in the study. Before starting the study, its purpose and methodology were explained, and participants were informed that participation was voluntary and that they could withdraw from the study at any time. Written and verbal consent was obtained from the participants. To eliminate the language barrier, communication with participants was established in Turkish and those who faced a language barrier were excluded from the study. Completion of the questionnaires took approximately 10 minutes.

### Data Analysis

The data were analyzed using SPSS (Statistical Package for Social Sciences) version 20. Descriptive statistics, including frequencies, percentages, and means, were calculated for the participants. For all analyses, the statistical significance threshold was set at p < 0.05. The mean total score of the scales was calculated, using the Shapiro-Wilk normality test to determine the normal distribution of the scale scores. Because the scale scores had a normal distribution, a One-Way ANOVA test was conducted to assess the significance of differences between the means of three or more groups and the Bonferroni test was used as a post-hoc test to identify which groups contributed to the significant differences. Independent t-test was used to compare the mean scores of two independent groups.

## RESULTS

Table 1 shows sociodemographic characteristics of the pregnant women.

**Table 1:** The sociodemographic characteristics of the pregnant women – Mardin, Türkiye, 2023.

Characteristics		n	%
Age	16–20 Age	20	13.7
	**21**–**25 Age**	**44**	**30.1**
	26–30 Age	43	29.5
	31–35 Age	25	17.1
	36 and ↑ Age	14	9.6
Obtaining citizenship	Yes	28	19.2
	**Not yet**	**118**	**80.8**
Place of living	Province	44	30.1
	**District**	**67**	**45.9**
	Village/town	35	24.0
Family type	Extended family	46	31.5
	**Nuclear family**	**100**	**68.5**
Education	Illiterate	42	28.8
	**Primary school graduate**	**62**	**42.5**
	Secondary education graduate	31	21.2
	Undergraduate and above	11	7.5
Husband’s education	Illiterate	32	21.9
	**Primary school graduate**	**67**	**45.9**
	Secondary education graduate	34	23.3
	Undergraduate and above	13	8.9
Employment status	Employed	12	8.2
	**Not employed**	**134**	**91.8**
Husband’s employment status	Employed	128	87.7
	**Not employed**	18	12.3
Income status	**Income less than expenditure**	**85**	**58.2**
	Income equal to expenditure	58	39.7
	Income more than expenditure	3	2.1
Housing type	Apartment	51	34.9
	**Detached House**	**95**	**65.1**
Social security	Have	31	21.2
	Do not have	**115**	**78.8**
Time lived in Türkiye	Less than 5 years	12	8.2
	**6**–**10 years**	**95**	**65.1**
	More than 10 years	39	26.7
Number of people in the household	**2**–**5 people**	**86**	**58.9**
	6-10 people	55	37.7
	11 and above	5	3.4
*Number of children under five years of age (N = 108)	1 child	46	42.6
	**2 children**	**47**	**43.5**
	3 children and above	15	13.9
Presence of chronic disease	Yes	18	12.3
	**No**	**128**	**87.7**
*Chronic Disease	Asthma	1	5.5
	Diabetes	2	11.2
	**Goiter**	**7**	**38.9**
	Kidney disease	1	5.5
	Hypertension	7	38.9
Total		**146**	**100**

*Percentages were calculated based on the answers given.

According to the table, their mean age is 27.2 ± 5.89, with 30.1% in the 21–25 age group. Of them, 80.8% have not yet obtained citizenship, 45.9% live in the district, and 68.5% have a nuclear family. Regarding educational levels, 42.5% of the pregnant women and 45.9% of their husbands are primary school graduates. Of the pregnant women, 91.8% are not employed, whereas this rate is 12.3% for their husbands, 58.2% have lower income than their expenses, 65.1% live in a detached house, and 78.8% do not have social security. Among them, 65.1% have lived in Turkey for 6–10 years. Of them, 58.9% report that the number of people in their household ranges from 2 to 5, 43.5% have two children under the age of 5 years, and 87.7% report no chronic illness. Among those with chronic illnesses (12.3%), 38.9% have goiter and the same percentage (38.9%) suffer from hypertension.

Table 2 presents the fertility and marital status of the pregnant women.

**Table 2:** The fertility and marital status of the pregnant women – Mardin, Türkiye, 2023.

Characteristics		n	%
Weeks of gestation	36.00 week	27	18.5
	37.00 week	34	23.3
	**38.00 week**	**44**	**30.1**
	39.00 week	30	20.5
	40.00 week	11	7.5
From whom the PC received	**Midwife/nurse**	**117**	**80.1**
	Doctor	29	19.9
Number of PC received	**< 4**	**131**	**89.7**
	≥ 4	15	10.3
*Reason for receiving less than 4 PC (N = 131)	Family causes	4	3.1
	**Ignorance**	**52**	**39.7**
	Financial inadequacy	35	26.7
	Health-related	3	2.3
	Transportation	23	17.5
	Lack of time	14	10.7
Number of pregnancies	First	25	17.1
	**2 and more**	**121**	**82.9**
Age of marriage	13–18 years old	58	39.7
	**19**–**25 years old**	**79**	**54.1**
	26 and above	9	6.2
How many years she has been married	**1**–**10 years**	**108**	**74.0**
	11–25 years	38	26.0
Age at first pregnancy	**13**–**18 years**	**46**	**31.5**
	19–25 years	81	55.5
	26 and above	19	13.0
Duration between the previous pregnancy (N = 119)	1 year	32	26.9
	**2 years**	**36**	**30.2**
	3 years	19	16.0
	4 years and above	32	26.9
*Number of Children (N = 119)	1 child	28	23.5
	2 children	30	25.2
	**3 children**	**31**	**26.1**
	4 children and above	30	25.2
Having health problems during this pregnancy (N = 146)	**Yes**	**24**	**16.4**
	No	122	83.6
*Problems experienced (N = 24)	Bleeding	4	16.6
	Edema	4	16.6
	**Diabetes**	**6**	**25.0**
	Vomiting	1	4.2
	Hypertension	4	16.6
	Hypotension	1	4.2
	Infection	1	4.2
	Developmental delay	1	4.2
	Risk of premature birth	2	8.4
Planned pregnancy	**Yes**	**103**	**70.5**
	No	43	29.5
Expected mode of delivery	**Vaginal delivery**	**93**	**63.7**
	Caesarean section	53	36.3
Preferred Mode of Delivery	**Vaginal delivery**	**101**	**69.2**
	Caesarean section	45	30.8
Fetal Gender	**Girl**	**74**	**50.7**
	Boy	55	37.7
	Unknown	17	11.6
Total		165	100.0

*Percentages were calculated based on the answers given.

Accordingly, 30.1% of the pregnant women were at 38 weeks of gestation, 82.9% had experienced two or more pregnancies, 54.1% married between the ages of 19–25, 31.5% had their first pregnancy between the ages of 13–18, and 30.2% had a 2-year duration between their previous pregnancy and the current one. Of them, 26.1% had three children, 16.4% reported experiencing health problems during this pregnancy, and among those with health problems, 25.0% stated that the health problem was diabetes.

The descriptive statistical distribution and Cronbach’s Alpha values of the PCSS and its subscales are presented in Table 3.

**Table 3:** The descriptive statistical distribution and Cronbach’a Alpha values of the PCSS and its subscales – Mardin, Türkiye, 2023.

Scale and subscales	N	Min.	Max.	Mean	Sd	Cronbach’s alpha (α)
*Prenatal Care Satisfaction Scale (PCSS)*	**146**	**35.00**	**104.00**	**73.39**	**14.78**	**0.93**
The art of care	146	7.00	25.00	16.69	3.75	0.81
Technical quality	146	7.00	24.00	17.27	3.70	0.75
Accessibility	146	3.00	15.00	9.11	2.66	0.71
Physical environment	146	5.00	20.00	13.06	3.21	0.72
Suitability	146	4.00	15.00	10.27	2.25	0.73

The mean score of the PCSS is 73.39 ± 14.78. In the present study, the Cronbach’s alpha coefficient was 0.93.

The comparison of the total and subscale scores of the PCSS with some characteristics of the pregnant women is presented in Table 4.

**Table 4:** The comparison of the total and subscale scores of the PCSS with some characteristics of the pregnant women – Mardin, Türkiye, 2023.

Characteristics		PCSS	The art of care	Technical quality	Accessibility	Physical environment	Suitability
Place of living*	Village/town^a^	69.71 ± 15.81	16.34 ± 3.84	16.22 ± 4.04	7.71 ± 2.85	12.42 ± 3.44	10.02 ± 2.18
	District^b^	75.95 ± 14.31	17.19 ± 3.61	17.88 ± 3.55	9.59 ± 2.41	13.77 ± 3.12	10.47 ± 2.23
	Province^c^	72.43 ± 14.20	16.22 ± 3.88	17.18 ± 3.52	9.50 ± 2.51	12.50 ± 2.99	10.15 ± 2.33
	**Statistical Analysis**	F = 2.221 P = 0.11	F = 1.087 P = 0.34	F = 1.087 P = 0.34	**F = 6.931** **P = 0.00**	**F = 3.098** **P = 0.04**	F = 0.539 P = 0.58
	**Difference**				a-b-c. a-b. a-c		
Fetal Gender*	Girl^a^	75.87 ± 13.26	17.24 ± 3.34	17.95 ± 3.25	9.44 ± 2.53	13.50 ± 3.057	10.64 ± 2.14
	Boy^b^	72.32 ± 14.56	16.65 ± 3.79	16.80 ± 3.64	9.07 ± 2.54	12.87 ± 3.055	9.90 ± 2.25
	Unknown^c^	66.05 ± 19.25	14.47 ± 4.66	15.82 ± 5.08	7.82 ± 3.26	11.82 ± 4.08	9.82 ± 2.50
	**Statistical Analysis**	**F = 3.39** **P = 0.03**	**F = 3.923** **P = 0.02**	F = 3.113 **P = 0. 04**	F = 2.639 P = 0.07	F = 2.078 P = 0.12	F = 2.131 P = 0.12
	**Difference**	a–c	a–c	a–c			
Income status*	Income less than expenditure	74.87 ± 14.49	16.84 ± 3.74	17.90 ± 3.58	9.25 ± 2.55	13.30 ± 3.22	10.49 ± 2.18
	Income equal to expenditure	71.79 ± 14.91	16.65 ± 3.69	16.55 ± 3.67	8.94 ± 2.81	12.79 ± 3.23	9.94 ± 2.35
	Income more than expenditure	62.66 ± 18.03	13.33 ± 5.03	13.33 ± 3.78	8.33 ± 3.51	11.66 ± 2.08	10.33 ± 1.15
	**Statistical Analysis**	F = 1.56 P = 0.21	F = 1.279 P = 0.28	F = 4.222 **P = 0. 01**	F = 0.364 P = 0.69	F = 0.729 P = 0.48	F = 1.020 P = 0.36
	**Difference**			a-c			
Time lived in Türkiye*	Less than 5 years^a^	76.16 ± 12.91	17.66 ± 3.55	17.75 ± 3.048	8.41 ± 3.28	14.08 ± 2.93	10.75 ± 2.37
	6–10 years^b^	71.78 ± 15.96	16.43 ± 4.00	16.93 ± 3.92	8.80 ± 2.76	12.78 ± 3.50	10.03 ± 2.33
	More than 10 years^c^	76.46 ± 11.66	17.05 ± 3.13	17.94 ± 3.26	10.10 ± 1.87	13.43 ± 2.42	10.71 ± 1.93
	**Statistical Analysis**	F = 1.625 P = 0.20	F = 30.808 P = 0.44	F = 1.143 P = 0.32	**F = 3.915** **P = 0.02**	F = 1.217 P = 0.29	F = 1.599 P = 0.20
	**Difference**				b-c		
Number of children under five years of age*	1 child^a^	75.54 ± 14.97	17.30 ± 3.98	17.95 ± 3.74	9.26 ± 2.74	13.23 ± 3.42	10.69 ± 2.22
	2 children^b^	73.50 ± 14.91	16.39 ± 3.80	17.10 ± 3.74	9.21 ± 2.39	13.28 ± 3.38	10.32 ± 2.10
	3 children and above^c^	62.93 ± 15.18	14.53 ± 3.83	14.60 ± 4.10	7.66 ± 2.60	11.20 ± 2.95	8.86 ± 2.41
	**Statistical Analysis**	**F = 4.073** **P = 0.20**	F = 2.915 P = 0.06	**F = 4.424** **P = 0.01**	F = 2.400 P = 0.09	F = 2.449 P = 0.09	**F = 3.909** **P = 0.02**
	**Difference**	a–c		a-c			a-c
From whom the PC received**	Midwife/nurse	74.52 ± 14.66	16.81 ± 3.80	17.59 ± 3.70	9.20 ± 2.57	13.39 ± 3.13	10.46 ± 2.09
	Doctor	68.86 ± 14.63	16.24 ± 3.62	16.00 ± 3.48	8.76 ± 3.02	11.76 ± 3.26	9.52 ± 2.71
	**Statistical Analysis**	t = 1.862 p = 0.06	t = 0.731 p = 0.46	**t = 2.094** **p = 0.04**	t = 0.808 p = 0.42	**t = 2.498** **p = 0.01**	**t = 2.050** **p = 0.04**
Number of PC received**	< 4	72.62 ± 14.92	16.56 ± 3.78	17.11 ± 3.73	8.86 ± 2.60	12.98 ± 3.30	10.19 ± 2.23
	≥ 4	80.20 ± 11.78	17.87 ± 3.40	18.67 ± 3.27	11.40 ± 2.13	13.80 ± 2.27	11.00 ± 2.30
	**Statistical Analysis**	t = -1.899 p = 0.06	t = -1.274 p = 0.20	t = -1.546 p = 0.12	**t = -3.655** **p = 0.00**	t = -1.246 p = 0.23	t = -1.326 p = 1.19
Planned pregnancy**	Yes	74.82 ± 14.45	17.18 ± 3.56	17.38 ± 3.67	9.43 ± 2.55	13.31 ± 3.17	10.38 ± 2.32
	No	69.97 ± 15.14	15.53 ± 3.99	17.00 ± 3.80	8.34 ± 2.78	12.48 ± 3.25	10.00 ± 2.03
	**Statistical Analysis**	t = 1.821 p = 0.07	**t = 0.479** **p = 0.01**	t = -1.695 p = 0.092	**t = -2.647** **p = 0.009**	t = 1.415 p = 0.159	t = 0.952 p = 0.343
Social security**	Have	77.48 ± 11.23	17.54 ± 3.09	17.90 ± 2.80	9.80 ± 2.28	13.93 ± 2.64	10.74 ± 1.86
	Do not have	72.29 ± 15.45	16.46 ± 3.89	17.10 ± 3.90	8.93 ± 2.73	12.83 ± 3.31	10.14 ± 2.32
	**Statistical Analysis**	**t = 2.092** **p = 0.04**	t = 1.424 p = 0.15	t = 0.576 p = 0.56	**t = 0.468** **p = 0.02**	t = 1.415 p = 0.15	t = 0.952 p = 0.34

*One Way Anova Test;

**Independent Samples T-Test.

According to the table, there is no significant difference in their scores of age, education, spouse’s education, number of people in the household, gestational week, marriage age, age at first pregnancy, marriage year, duration since the previous pregnancy, number of children, citizenship status, employment status of the women and their spouses, type of residence, family type, chronic diseases, pregnancy-related problems, and expected and preferred birth method (p > 0.05). However, there is a significant correlation between the place of residence and the physical environment and accessibility (p < 0.05). The correlation is significant between fetal gender and the total score of the PCSS, as well as the subscales of care quality and technical quality (p < 0.05). There is a significant difference between duration of residence in Turkey and the accessibility subscale (p < 0.05). There is a significant difference between the number of children under the age of five and the total score of the PCSS, as well as the technical quality and accessibility subscales (p < 0.05). There is a significant difference between income status and the technical quality subscales (p < 0.05).

## DISCUSSION

Pregnant refugees and disadvantaged women are considered a vulnerable group. This group is at a higher risk of serious neonatal and maternal health issues due to factors such as increased cesarean risk, inadequate primary health conditions, and more complex pregnancy processes^(19)^.

In the study, the most common reasons for Syrian pregnant women not receiving sufficient PC are “ignorance” (39.7%), “financial difficulty” (26.7%), and “transportation issues” (17.5%). The finding related to ignorance aligns with literature suggesting that a lack of knowledge about PC services prevents regular attendance at check-ups^(20)^. In particular, the lack of awareness about the importance of healthcare is significant for migrant groups. This may hinder migrant women from managing their pregnancy more consciously and using healthcare services regularly. Additionally, previous studies indicate that migrant women generally report lower satisfaction with prenatal care services due to factors such as delayed access, insufficient information, and communication challenges with healthcare professionals^(10)^. Similar to our findings, these barriers often result in inconsistent or inadequate prenatal care.

The second most common reason is “financial difficulty” that can be related to the majority of the participants (58.2%) having lower income than their expenses. The findings of our study highlight the challenges faced in accessing healthcare services, particularly due to economic difficulties. The group with income lower than expenses has a higher mean score for the “technical quality” subscale, which reflects satisfaction primarily due to healthcare personnel, compared to the group with higher income. In a study involving disadvantaged women, barriers to accessing healthcare services included issues such as the change of healthcare personnel each time, their disrespectful behaviors, not considering patients’ needs, not dedicating enough time for PC, and being inconsiderate^(21)^. In another study, conversely, the satisfactory aspects of healthcare services included well-equipped hospitals (56.1%), the positive attitude of doctors and healthcare personnel (55.1%), and the competence of doctors and healthcare personnel (46.2%)^(22)^. Compared to the literature, the results of the present study suggest that the provision of regular and free standard healthcare services in public hospitals for migrants who have acquired citizenship or temporary protection identification cards in Turkey^(23,24)^ is satisfactory for those with low income but insufficient to meet the needs of those with higher income (e.g., queues, waiting periods, physical environment, etc.).

Transportation issues are another significant factor restricting access to PC services for Syrian pregnant women. The rate of those receiving fewer than four PC visits is 89.7% and the accessibility subscale score is lower among these individuals, which clearly indicates transportation barriers. In the study by Penman et al.^(21)^, the distance between home and the hospital, as well as the availability or solidity of public transportation, were identified as barriers to accessing healthcare services. In our study, 45.9% of the participants lived in rural areas, which may have prevented them from continuing full care at the hospital located in the city. Another finding from the study is that 78.8% of the participants did not have social security, which may have also contributed to this outcome. The literature also suggests that having social security not only facilitates access to healthcare services but also increases satisfaction with these services^(10,25)^. In the present study, because 80.8% of Syrian pregnant women have not yet obtained citizenship, they may not be well informed about free access to healthcare services with a temporary identity card.

The presence of children under the age of 5 (73.97%) can also indirectly affect the number of follow-up visits. In our study, those with one child under 5 years old had higher PC satisfaction, technical quality, and suitability scores. In the study by Penman et al.^(21)^, all women mentioned social issues (lack of social support from family or friends, family responsibilities, etc.) as barriers to accessing PC or receiving care. Regarding family responsibilities and household chores, women stated that taking care of other children sometimes prevents them from attending appointments, and when they bring their children to PC appointments, they are not well received by healthcare personnel. In a study where women mentioned that the number of children is part of their culture, they stated receiving negative reactions for this and found it disturbing^(26)^. The study results are consistent with the literature. Having more children, as part of their culture, and the lack of sufficient social support may have affected the satisfaction with hospital care for Syrian pregnant women, especially when they are exposed to reactions from healthcare personnel when presenting at the hospital with their children.

They stated that they received 80.1% of their PC from midwives/nurses. There is a significant difference in the mean scores for the subscales of technical quality, physical environment, and suitability between those who received PC from midwives/nurses and those who received it from doctors. Those who received care from midwives/nurses obtained higher scores. In a study conducted in Italy, pregnant women indicated trusted midwives the most, with a rate of 81.2%^(27)^. The presence of a similar result suggests that midwives’ empathy and spiritual care had a positive effect on women’s satisfaction (technical quality and suitability). In a study conducted in Norway, however, there was no significant difference in terms of dissatisfaction between women receiving birth care from practitioners, midwives, or obstetricians^(10)^. The evaluation of Syrian pregnant women in the present study may be explained by the differences in cultural structures.

In our study, 70.5% of participants stated that their pregnancies were planned. Those with planned pregnancies had higher scores in the art of care and accessibility subscales. The literature also emphasizes that planned pregnancies have a positive effect on satisfaction with healthcare services^(28)^. In the current study, the pregnant women’s satisfaction score made up more than half of the total score. However, the satisfaction derived from the service is possibly increased due to the significance of planned pregnancies for the family and that healthcare is free for migrants with identification cards.

## STUDY LIMITATIONS

- This study is limited to Syrian pregnant women who applied to Mardin Training and Research Hospital. Therefore, the results cannot be generalized to the overall Syrian refugee population.
- The research was conducted between August 15, 2023, and September 16, 2023. Longer-term studies may yield different results.
- Since participants were selected using a purposive sampling method, random sampling was not performed, limiting the generalizability of the findings.
- Only Syrian pregnant women who could speak Turkish were included in the study. This may have led to the exclusion of women who face greater challenges in accessing healthcare services due to language barriers.
- Participants’ satisfaction with healthcare services may have been influenced by cultural norms, which should be taken into consideration.

## CONCLUSION

The study findings indicate that lack of information, economic difficulties, lack of social security, and transportation issues are major barriers to healthcare access for migrant pregnant women.

Factors such as receiving PC from midwives or nurses, having planned pregnancies, and having social security influenced PC satisfaction of pregnant women in the study. These results shed light on the development of recommendations to improve satisfaction with PC. Based on these results:

- Migrant women should receive information about the importance of PC services.
- Healthcare personnel should receive training on empathy and cultural sensitivity towards migrant women.
- Free transportation local support should be provided to reduce transportation-related problems.
- The scope of social security should be expanded to enable more migrant women to benefit from free healthcare services.
- Further studies in different regions with larger sample sizes could further enrich the knowledge base in this subject.
