# Supplementary material for: An evaluation of the Syrian pregnant women’s prenatal care satisfaction: a cross-sectional study
Source: Rev Esc Enferm USP. 2025 May 16;59:e20250002. doi: 10.1590/1980-220X-REEUSP-2025-0002en (PMC12083561; doi:10.1590/1980-220X-REEUSP-2025-0002en)
Supplement: Translation

# Translation: Avaliação da satisfação de gestantes sírias com os cuidados pré-natais: um estudo transversal*

**DOI:** 10.1590/1980-220X-REEUSP-2025-0002pt

**Authors:** Sibel İçke, Sema Çifçi

**Affiliations:** 1 Mardin Artuklu University, Faculty of Health Sciences, Midwifery Department, Artuklu, Mardin, Türkiye.; 2 Mardin Artuklu University, Faculty of Health Sciences, Nursing Department, Artuklu Mardin, Türkiye.

**Keywords:** Migrantes, Gestantes, Cuidado Pré-Natal, Satisfação Pessoal

## Abstract

**Objetivo:**

O objetivo deste estudo é examinar os níveis de satisfação das gestantes migrantes sírias que vivem em Mardin com os serviços de atendimento pré-natal e os fatores que influenciam sua satisfação.

**Método:**

Estudo transversal em que a população foi composta por gestantes sírias que se inscreveram no Mardin Training and Research Hospital entre 15 de agosto e 16 de setembro de 2023. Participaram do estudo 146 gestantes sírias que atenderam aos critérios de inclusão. O formulário de informações sociodemográficas e a Escala de Satisfação com o Atendimento Pré-Natal (ESAPN) foram usados como ferramentas de coleta de dados.

**Resultados:**

A taxa de gestantes que receberam atendimento pré-natal de uma parteira ou enfermeira foi de 80,1% e a das que receberam menos de quatro atendimentos pré-natais foi de 89,7%. O motivo mais comum para não receber assistência pré-natal adequada foi a falta de informações, com taxa de 39,7%. A pontuação média da ESAPN foi de 73,39 ± 14,78.

**Conclusões:**

Os resultados do estudo indicam que a falta de informação é uma das principais barreiras ao acesso à assistência à saúde para as gestantes migrantes. Além disso, o fato de receber serviços de atendimento pré-natal de parteira sou enfermeiras afetou a satisfação com o atendimento pré-natal.

## INTRODUÇÃO

O cuidado pré-natal (CP) refere-se aos serviços de saúde prestados por profissionais de saúde às gestantes durante toda a gestação para monitorar e manter a saúde ideal da mãe e do bebê^(1,2)^. O CP de alta qualidade é crucial, pois incorpora intervenções de promoção da saúde para a mãe e o feto, identifica potenciais condições adversas em um estágio inicial e facilita intervenções oportunas^(2)^. Embora as taxas globais de mortalidade materna tenham diminuído aproximadamente 34% entre 2000 e 2020, essas taxas permanecem significativamente altas em países de baixa e média renda, respondendo por 95% de todas as mortes maternas^(3)^.

A guerra civil que teve início na Síria em 2011 levou à recessão econômica^(4,5)^ e desencadeou a migração generalizada. A Turquia, sem dúvida, tem sido o país mais afetado pela migração de sírios^(5,6)^. Desde 2024, o número de sírios sob proteção temporária na Turquia é de aproximadamente 3,1 milhões, dos quais quase 1,5 milhão são mulheres e aproximadamente 725.000 têm idade entre 15 e 49 anos^(7)^.

Ao se mudarem para outros países, os migrantes sírios enfrentam vários desafios e um dos principais problemas são as questões relacionadas à saúde^(8)^. A falta de acesso ao CP é um desses desafios. Ser migrante é um dos fatores que influenciam o acesso ao CP. As mulheres migrantes beneficiam-se menos dos serviços de Atenção Básica (AB) devido a barreiras como idioma, educação e transporte^(9,10)^. De acordo com os dados da Pesquisa Demográfica e de Saúde da Turquia de 2018, 11% dos migrantes sírios não recebem nenhum CP e 33,8% recebem cuidados insuficientes, significativamente menores em comparação com as mulheres grávidas nativas^(11)^. Em pesquisa realizado com mulheres refugiadas sírias, a taxa das que não receberam CP foi de 41,3%, enquanto entre as cidadãs turcas foi de 7,7%^(12)^.

A Organização Mundial da Saúde (OMS) recomenda pelo menos oito consultas pré-natais durante a gestação. No entanto, a cobertura global de assistência pré-natal ainda não está em um nível suficiente. De acordo com os dados da OMS e das Estatísticas Mundiais de Saúde de 2016, apenas 64% das mulheres em todo o mundo receberam CP quatro ou mais vezes durante a gravidez^(13)^.

A satisfação é crucial em todas as áreas da saúde, incluindo os CP^(9)^. Estudos revelam que mulheres migrantes relatam menor satisfação com os serviços de saúde devido ao atraso, informação insuficiente e desafios de comunicação^(14–16)^. A insatisfação com os serviços de AB pode levar a dificuldades de adaptação aos sistemas de saúde do país de destino.

O presente estudo tem como objetivo examinar os níveis de satisfação de mulheres grávidas migrantes sírias que vivem em Mardin, Turquia, com os serviços de CP e os fatores que influenciam sua satisfação.

Questões de pesquisa:

1. Quais são os níveis de satisfação de mulheres grávidas sírias com os serviços de PC?
2. Que fatores influenciam os níveis de satisfação de gestantes sírias com os serviços de CP?

## MÉTODO

### Tipo de Pesquisa

Trata-se de um estudo transversal.

### População e Amostra

A população do estudo foi composta por mulheres sírias grávidas que visitaram o Hospital de Treinamento e Pesquisa de Mardin entre 15 de agosto e 16 de setembro de 2023. Como o estudo foi realizado exclusivamente com mulheres migrantes sírias grávidas internadas no hospital, as participantes foram selecionadas usando o método de amostragem intencional. Participaram do estudo 146 gestantes sírias que atenderam aos critérios de inclusão.

Para avaliar o poder estatístico do estudo, foi realizada análise de poder post-hoc para o teste t de uma amostra usando o software G*Power 3.1. Na análise, foram utilizados um efeito de tamanho moderado (d = 0,5), nível de significância estatística (α = ٠,٠٥) e tamanho total da amostra (n = 146). O poder obtido foi calculado como (1-β) = 0,9999. Este resultado mostra que o estudo tem poder estatístico bastante alto para detectar um tamanho de efeito moderado.

### Critério de Inclusão

1. Ser síria,
2. Período gestacional entre 36 e 40 semanas,
3. Não ter barreiras linguísticas.

### Critério de Exclusão

1. Não ser síria,
2. Período gestacional fora do intervalo de 36 a 40 semanas,
3. Ter barreira linguística.

### Ferramentas de Coleta de Dados

O estudo utilizou um questionário composto por duas seções: a primeira coletou informações sociodemográficas, enquanto a segunda incluiu a Escala de Satisfação com o Cuidado Pré-natal (ESCP), cuja validade e confiabilidade turcas foram avaliadas por Aslantekin Özçoban et al.^(17)^ e publicadas em 2020.

***- Ficha de Identificação Individual (IFI):*** Desenvolvida pelos pesquisadores com base na literatura, contém informações sociodemográficas e obstétricas. Composta por 36 questões.

***- Escala de Satisfação com o Cuidado Pré-natal (PCSS):*** desenvolvida originalmente por Raube et al.^(18)^ em 1998 para medir a satisfação das gestantes com a AB^(17,18)^, foi adaptada para o turco e validada quanto à confiabilidade por Aslantekin Özçoban et al.^(17)^ em 2020. Trata-se de uma escala do tipo Likert autorreferida composta por 22 itens. Com base na literatura, avalia cinco subescalas: arte de cuidar, qualidade técnica, ambiente físico, acessibilidade e adequação.

*Subescala Arte de Cuidar:* Inclui o valor percebido sobre os serviços recebidos em relação ao custo/prêmio pago, a capacidade da equipe de proporcionar conforto e sensação de segurança, a atenção de enfermeiras, parteiras e médicos, o comportamento respeitoso dos profissionais de saúde, a empatia e a compreensão demonstradas pela equipe da recepção durante estados emocionais como ansiedade, excitação ou raiva. Também avalia o respeito da equipe de recepção e o interesse e a abordagem demonstrados pela equipe de boas-vindas/registro.

*Subescala de Qualidade Técnica:* Inclui a adequação das explicações fornecidas por enfermeiras, parteiras e médicos sobre procedimentos, sua competência na prestação de cuidados e gerenciamento do parto, a atenção aos exames médicos e a adequação de suprimentos e equipamentos de saúde.

*Subescala de Acessibilidade:* Avalia as condições de transporte, o período de espera para exames durante a consulta, a duração entre a data da consulta inicial e a data agendada para o próximo acompanhamento e a adequação do horário de funcionamento do serviço.

*Subescala de Ambiente Físico:* Avalia a limpeza, o conforto e a conveniência das instalações, a condição das áreas de exame e a adequação das salas de espera.

*Subescala de Adequação:* Inclui o tempo alocado pela equipe que fornece aconselhamento nutricional durante a gravidez, a competência profissional de enfermeiras, parteiras e médicos e a viabilidade do aconselhamento fornecido por profissionais de saúde durante a gravidez para proteger a saúde da mãe e do bebê.

Os valores do alfa de Cronbach para as subescalas variam de 0,7 a 0,9 e é de 0,95 para a escala geral. Não há ponto de corte na escala. Neste estudo, a pontuação mais baixa foi 22, enquanto a mais alta foi 110. As respostas na escala são classificadas da seguinte forma: ruim (1), regular (2), bom (3), muito bom (4) e excelente (5). À medida que a pontuação na escala aumenta, a satisfação é considerada mais alta e, à medida que a pontuação diminui, a satisfação é considerada mais baixa^(17)^.

### Considerações Éticas

Antes do estudo, foi obtida a aprovação do Comitê de Ética em Pesquisa Clínica Não Intervencionista da Universidade Mardin Artuklu com o número de decisão 2022-9, datada de 01/08/2022. O apoio financeiro para o estudo foi fornecido pelo projeto MAÜ. BAP.22.SBF.026, destinada à realização da pesquisa pela Coordenação de Projetos de Pesquisa Científica da Universidade Mardin Artuklu.

### Coleta de Dados

Os dados do estudo foram coletados por meio de pesquisa face a face nos ambulatórios de obstetrícia e ginecologia, no ambulatório de teste não ergométrico e nas aulas de pré-natal. Os questionários foram realizados em turco com as gestantes que concordaram em participar. Antes de iniciar o estudo, seu objetivo e metodologia foram explicados e os participantes foram informados de que a participação era voluntária e que poderiam desistir a qualquer momento. Foi obtido o consentimento verbal e por escrito das participantes. Para eliminar a barreira do idioma, a comunicação com as participantes foi estabelecida em turco e aquelas que enfrentaram barreira linguística foram excluídas. O preenchimento dos questionários levou aproximadamente 10 minutos.

### Análise de Dados

Os dados foram analisados por meio do programa SPSS (*Statistical Package for Social Sciences*) versão 20. Estatísticas descritivas, incluindo frequências, porcentagens e médias, foram calculadas para as participantes. Em todas as análises, o limiar de significância estatística foi estabelecido em p < 0,05. A pontuação total média das escalas foi calculada utilizando-se o teste de normalidade de Shapiro-Wilk para determinar a distribuição normal dos escores das escalas. Como tais escores tinham distribuição normal, um teste ANOVA One-Way foi realizado para avaliar a significância das diferenças entre as médias de três ou mais grupos e o teste de Bonferroni foi usado como um teste post-hoc para identificar quais grupos contribuíram para as diferenças significativas. O teste t independente foi usado para comparar os escores médios de dois grupos independentes.

## RESULTADOS

A Tabela 1 apresenta as características sociodemográficas das gestantes.

**Tabela 1:** Características sociodemográficas das gestantes – Mardin, Turquia, 2023.

Características		n	%
Idade	16–20 Idade	20	13,7
	**21**–**25 Idade**	**44**	**30,1**
	26–30 Idade	43	29,5
	31–35 Idade	25	17,1
	36 e ↑ Idade	14	9,6
Obtenção da cidadania	Sim	28	19,2
	**Ainda não**	**118**	**80,8**
Local de residência	Província	44	30,1
	**Distrito**	**67**	**45,9**
	Vila/cidade	35	24,0
Tipo de família	Família extensa	46	31,5
	**Família nuclear**	**100**	**68,5**
Educação	Analfabeto	42	28,8
	**Graduado do ensino fundamental**	**62**	**42,5**
	Pós-graduado do ensino médio	31	21,2
	Graduação e acima	11	7,5
Educação do marido/companheiro	Analfabeto	32	21,9
	**Graduado do ensino fundamental**	**67**	**45,9**
	Pós-graduado do ensino médio	34	23,3
	Graduação e acima	13	8,9
Situação professional	Empregado	12	8,2
	**Não empregado**	**134**	**91,8**
Situação de emprego do marido	Empregado	128	87,7
	**Não empregado**	18	12,3
Renda	**Receitas inferiores** às**despesas**	**85**	**58,2**
	Receitas iguais às despesas	58	39,7
	Receitas maiores que despesas	3	2,1
Tipo de Moradia	Apartamento	51	34,9
	**Moradia Isolada**	**95**	**65,1**
Segurança social	Tem	31	21,2
	Não tem	**115**	**78,8**
O tempo vivido na Turquia	Menos de 5 anos	12	8,2
	**6**–**10 anos**	**95**	**65,1**
	Mais de 10 anos	39	26,7
Número de pessoas na família	**2**–**5 pessoas**	**86**	**58,9**
	6–10 pessoas	55	37,7
	11 e acima	5	3,4
*Número de crianças menores de cinco anos (N = 108)	1 criança	46	42,6
	**2 crianças**	**47**	**43,5**
	3 crianças e acima	15	13,9
Presença de doença crônica	Sim	18	12,3
	**Não**	**128**	**87,7**
*Doença Crônica	Asma	1	5,5
	Diabetes	2	11,2
	**Bócio**	**7**	**38,9**
	Doença renal	1	5,5
	Hipertensão	7	38,9
Total		**146**	**100**

*As porcentagens foram calculadas com base nas respostas dadas.

De acordo com a tabela, a idade média foi de 27,2 ± 5,89, com 30,1% na faixa etária de 21 a 25 anos. Destas, 80,8% ainda não obtido a cidadania, 45,9% moravam no distrito, 68,5% em famílias nucleares. Em relação à escolaridade, 42,5% das gestantes e 45,9% dos maridos eram formados no ensino fundamental. Das gestantes, 91,8% não estavam empregadas, enquanto essa taxa era de 12,3% para seus maridos, 58,2% tinham renda inferior às despesas, 65,1% moravam em casa isolada e 78,8% não possuíam previdência social. Entre elas, 65,1% viviam na Turquia entre 6 e 10 anos. Destas, 58,9% relataram que o número de pessoas em seu domicílio variava de 2 a 5, 43,5% tinham dois filhos menores de 5 anos e 87,7% não relataram doenças crônicas. Entre aquelas com doenças crônicas (12,3%), 38,9% apresentavam bócio e o mesmo percentual (38,9%), hipertensão.

A Tabela 2 apresenta a fecundidade e o estado civil das gestantes.

**Tabela 2:** Fertilidade e estado civil das mulheres grávidas – Mardin, Turquia, 2023.

Características		n	%
Semanas de gestação	36 semanas	27	18,5
	37 semanas	34	23,3
	**38 semanas**	**44**	**30,1**
	39 semanas	30	20,5
	40 semanas	11	7,5
De quem recebeu CP?	**Parteira/enfermeira**	**117**	**80,1**
	Médico	29	19,9
Número de consultas recebido	**< 4**	**131**	**89,7**
	≥ 4	15	10,3
*Motivo para receber menos de 4 consultas (N = 131)	Causas familiares	4	3,1
	**Desconhecimento**	**52**	**39,7**
	Inadequação financeira	35	26,7
	Relacionadas com a saúde	3	2,3
	Transporte	23	17,5
	Falta de tempo	14	10,7
Número de gestações	Primeiro	25	17,1
	**2 e mais**	**121**	**82,9**
Tempo de casamento	13–18 anos	58	39,7
	**19**–**25 anos**	**79**	**54,1**
	26 e acima	9	6,2
Há quantos anos está casada?	**1**–**10 anos**	**108**	**74,0**
	11–25 anos	38	26,0
Idade da primeira gravidez	**13**–**18 anos**	**46**	**31,5**
	19–25 anos	81	55,5
	26 e acima	19	13,0
Duração entre a gravidez anterior (N = 119)	1 ano	32	26,9
	**2 anos**	**36**	**30,2**
	3 anos	19	16,0
	4 anos ou mais	32	26,9
*Número de filhos (N = 119)	1 criança	28	23,5
	2 crianças	30	25,2
	**3 crianças**	**31**	**26,1**
	4 crianças e acima	30	25,2
Problemas de saúde durante esta gravidez (N = 146)	**Sim**	**24**	**16,4**
	Não	122	83,6
*Problemas experimentados (N = 24)	Hemorragia	4	16,6
	Edema	4	16,6
	**Diabetes**	**6**	**25,0**
	Vômito	1	4,2
	Hipertensão	4	16,6
	Hipotensão	1	4,2
	Infeção	1	4,2
	Atraso no desenvolvimento	1	4,2
	Risco de parto prematuro	2	8,4
Gravidez planejada	**Sim**	**103**	**70,5**
	Não	43	29,5
Modo de entrega esperado	**Parto vaginal**	**93**	**63,7**
	Cesariana	53	36,3
Modo de entrega preferido	**Parto vaginal**	**101**	**69,2**
	Cesariana	45	30,8
Sexo fetal	**Menina**	**74**	**50,7**
	Menino	55	37,7
	Desconhecido	17	11,6
Total		165	100,0

*As porcentagens foram calculadas com base nas respostas dadas.

Assim, 30,1% das gestantes estavam na 38^a^ semana de gestação, 82,9% tiveram duas ou mais gestações, 54,1% casaram-se entre 19 e 25 anos, 31,5% tiveram sua primeira gravidez entre 13 e 18 anos e 30,2% tiveram um espaçamento de dois anos entre a gravidez anterior e a atual. Destas, 26,1% tiveram três filhos, 16,4% relataram ter tido problemas de saúde durante a gravidez e, entre as que apresentavam problemas de saúde, 25,0% mencionaram o diabetes.

A estatística descritiva e os valores do Alfa de Cronbach da ESCP e suas subescalas são apresentados na Tabela 3.

**Tabela 3:** Distribuição estatística descritiva, valores de Alfa de *Cronbach da Escala de Satisfação com o Pré-Natal* e suas subescalas – Mardin, Turquia, 2023.

Escala e subescalas	N	Min.	Max.	Significar	Sd	Alfa de Cronbach (α)
*Escala de Satisfação com o Pré-Natal (PCSS)*	146	35,00	104,00	73,39	14,78	0,93
A arte de cuidar	146	7,00	25,00	16,69	3,75	0,81
Qualidade técnica	146	7,00	24,00	17,27	3,70	0,75
Acessibilidade	146	3,00	15,00	9,11	2,66	0,71
Ambiente físico	146	5,00	20,00	13,06	3,21	0,72
Adequabilidade	146	4,00	15,00	10,27	2,25	0,73

A pontuação média do PCSS é de 73,39 ± 14,78. No presente estudo, o coeficiente alfa de Cronbach foi de 0,93.

A comparação dos escores total e das subescalas do PCSS com algumas características das gestantes pode ser observada na Tabela 4.

**Tabela 4:** A comparação dos escores totais e das subescalas do PCSS com algumas características das gestantes – Mardin, Turquia, 2023.

Características		Referências	A arte de cuidar	Qualidade técnica	Acessibilidade	Ambiente físico	Adequabilidade
Local de residência*	Vila/cidade^a^	69,71 ± 15,81	16,34 ± 3,84	16,22 ± 4,04	7,71 ± 2,85	12,42 ± 3,44	10,02 ± 2,18
	Distrito B	75,95 ± 14,31	17,19 ± 3,61	17,88 ± 3,55	9,59 ± 2,41	13,77 ± 3,12	10,47 ± 2,23
	Província	72,43 ± 14,20	16,22 ± 3,88	17,18 ± 3,52	9,50 ± 2,51	12,50 ± 2,99	10,15 ± 2,33
	**Análise estatística**	F = 2,221 P = 0,11	F = 1,087 P = 0,34	F = 1,087 P = 0,34	**F = 6,931** **P = 0,00**	**F = 3,098** **P = 0,04**	F = 0,539 P = 0,58
	**Diferença**				a-b-c. a-b. a–c		
Sexo fetal*	Menina	75,87 ± 13,26	17,24 ± 3,34	17,95 ± 3,25	9,44 ± 2,53	13,50 ± 3,057	10,64 ± 2,14
	Menino	72,32 ± 14,56	16,65 ± 3,79	16,80 ± 3,64	9,07 ± 2,54	12,87 ± 3,055	9,90 ± 2,25
	Desconhecidoc	66,05 ± 19,25	14,47 ± 4,66	15,82 ± 5,08	7,82 ± 3,26	11,82 ± 4,08	9,82 ± 2,50
	**Análise estatística**	**F = 3,39** **P = 0,03**	**F = 3,923** **P = 0,02**	F = 3,113 **P = 0,04**	F = 2,639 P = 0,07	F = 2,078 P = 0,12	F = 2,131 P = 0,12
	**Diferença**	a–c	a–c	a–c			
Renda*	Receitas inferiores às despesas	74,87 ± 14,49	16,84 ± 3,74	17,90 ± 3,58	9,25 ± 2,55	13,30 ± 3,22	10,49 ± 2,18
	Receitas iguais às despesas	71,79 ± 14,91	16,65 ± 3,69	16,55 ± 3,67	8,94 ± 2,81	12,79 ± 3,23	9,94 ± 2,35
	Receitas maiores que as despesas	62,66 ± 18,03	13,33 ± 5,03	13,33 ± 3,78	8,33 ± 3,51	11,66 ± 2,08	10,33 ± 1,15
	**Análise estatística**	F = 1,56 P = 0,21	F = 1,279 P = 0,28	F = 4,222 **P = 0,01**	F = 0,364 P = 0,69	F = 0,729 P = 0,48	F = 1,020 P = 0,36
	**Diferença**			a–c			
Tempo vivido na Turquia*	Menos de 5 anos^a^	76,16 ± 12,91	17,66 ± 3,55	17,75 ± 3,048	8,41 ± 3,28	14,08 ± 2,93	10,75 ± 2,37
	6-10 anosb	71,78 ± 15,96	16,43 ± 4,00	16,93 ± 3,92	8,80 ± 2,76	12,78 ± 3,50	10,03 ± 2,33
	Mais de 10 anos^c^	76,46 ± 11,66	17,05 ± 3,13	17,94 ± 3,26	10,10 ± 1,87	13,43 ± 2,42	10,71 ± 1,93
	Istatistiksel Analiz	F = 1,625 P = 0,20	F = 30,808 P = 0,44	F = 1,143 P = 0,32	**F = 3,915** **P = 0,02**	F = 1,217 P = 0,29	F = 1,599 P = 0,20
	**Diferença**				b–c		
Número de crianças menores de cinco anos*	1 criança^a^	75,54 ± 14,97	17,30 ± 3,98	17,95 ± 3,74	9,26 ± 2,74	13,23 ± 3,42	10,69 ± 2,22
	2 crianças^b^	73,50 ± 14,91	16,39 ± 3,80	17,10 ± 3,74	9,21 ± 2,39	13,28 ± 3,38	10,32 ± 2,10
	3 crianças e acima^c^	62,93 ± 15,18	14,53 ± 3,83	14,60 ± 4,10	7,66 ± 2,60	11,20 ± 2,95	8,86 ± 2,41
	**Análise estatística**	**F = 4,073** **P = 0,20**	F = 2,915 P = 0,06	**F = 4,424** **P = 0,01**	F = 2,400 P = 0,09	F = 2,449 P = 0,09	**F = 3,909** **P = 0,02**
	**Diferença**	a–c		a–c			a–c
De quem o PC recebeu**	Parteira/enfermeira	74,52 ± 14,66	16,81 ± 3,80	17,59 ± 3,70	9,20 ± 2,57	13,39 ± 3,13	10,46 ± 2,09
	Médico	68,86 ± 14,63	16,24 ± 3,62	16,00 ± 3,48	8,76 ± 3,02	11,76 ± 3,26	9,52 ± 2,71
	**Análise estatística**	t = 1,862 p= 0,06	t = 0,731 p = 0,46	**t = 2,094** **p = 0,04**	t = 0,808 p = 0,42	**t = 2,498** **p = 0,01**	**t = 2,050** **p = 0,04**
Número de PCs recebidos**	< 4	72,62 ± 14,92	16,56 ± 3,78	17,11 ± 3,73	8,86 ± 2,60	12,98 ± 3,30	19/10 ± 2/23
	≥ 4	80,20 ± 11,78	17,87 ± 3,40	18,67 ± 3,27	11,40 ± 2,13	13,80 ± 2,27	11,00 ± 2,30
	**Análise estatística**	t = –1,899 p = 0,06	t = –1,274 p = 0,20	t = –1,546 p = 0,12	**t = –3,655** **p = 0,00**	t = –1,246 p = 0,23	t = –1,326 p = 1,19
Gravidez planejada**	Sim	74,82 ± 14,45	17,18 ± 3,56	17,38 ± 3,67	9,43 ± 2,55	13,31 ± 3,17	10,38 ± 2,32
	Não	69,97 ± 15,14	15,53 ± 3,99	17,00 ± 3,80	8,34 ± 2,78	12,48 ± 3,25	10,00 ± 2,03
	**Análise estatística**	t = 1,821 p = 0,07	**t = 0,479** **p = 0,01**	t = –1,695 p = 0,092	**t = –2,647** **p = 0,009**	t = 1,415 p = 0,159	t = 0,952 p = 0,343
Segurança social**	Tem	77,48 ± 11,23	17,54 ± 3,09	17,90 ± 2,80	9,80 ± 2,28	13,93 ± 2,64	10,74 ± 1,86
	Não tem	72,29 ± 15,45	16,46 ± 3,89	17,10 ± 3,90	8,93 ± 2,73	12,83 ± 3,31	10,14 ± 2,32
	**Análise estatística**	**t = 2,092** **p = 0,04**	t = 1,424 p = 0,15	t = 0,576 p = 0,56	**t = 0,468** **p = 0,02**	t = 1,415 p = 0,15	t = 0,952 p = 0,34

*Teste Anova de uma via;

**Teste T de amostras independentes.

De acordo com a tabela, não houve diferença significativa em suas pontuações de idade, educação, educação do cônjuge, número de pessoas na casa, semana gestacional, idade de casamento, idade da primeira gravidez, anos de casamento, espaçamento da gravidez anterior, número de filhos, status de cidadania, status de emprego das mulheres e seus cônjuges, tipo de residência, tipo de família, doenças crônicas, problemas relacionados à gravidez e tipo de parto esperado e preferido (p > 0,05). No entanto, observou-se correlação significativa entre o local de residência e o ambiente físico e a acessibilidade (p < 0,05), entre o sexo fetal e o escore total da ESCP, bem como as subescalas de qualidade assistencial e qualidade técnica (p < 0,05). Também houve diferença significativa entre o tempo de residência na Turquia e a subescala de acessibilidade (p < 0,05), entre o número de crianças menores de cinco anos e o escore total da ESCP, bem como as subescalas de qualidade técnica e acessibilidade (p < 0,05) e entre a renda e as subescalas de qualidade técnica (p < 0,05).

## DISCUSSÃO

Refugiadas grávidas e mulheres desfavorecidas são consideradas um grupo vulnerável que apresenta maior risco de problemas graves de saúde neonatal e materna devido a fatores como aumento do risco de cesarianas, condições inadequadas de saúde primária e processos gestacionais mais complexos^(19)^.

No presente estudo, as razões mais comuns para as mulheres grávidas sírias não receberem CP suficientes foram “desconhecimento” (39,7%), “dificuldade financeira” (26,7%) e “problemas de transporte” (17,5%). O achado relacionado ao desconhecimento está de acordo com a literatura, sugerindo que a falta de informação sobre os serviços de AB impede o comparecimento regular às consultas^(20)^. Em particular, a falta de conscientização sobre a importância dos cuidados de saúde é significativa para os grupos de migrantes. Isso pode impedir que as mulheres migrantes gerenciem sua gravidez de forma mais consciente e usem os serviços de saúde regularmente. Além disso, estudos anteriores indicam que as mulheres migrantes geralmente relatam menor satisfação com os serviços de pré-natal devido a fatores como acesso tardio, informação insuficiente e desafios de comunicação com os profissionais de saúde^10^. Essas barreiras geralmente resultam em cuidados pré-natais inconsistentes ou inadequados.

O segundo motivo mais comum é a “dificuldade financeira” que pode estar relacionada ao fato de a maioria das participantes (58,2%) ter renda inferior às despesas. Os achados do estudo destacaram os desafios enfrentados no acesso aos serviços de saúde, principalmente devido às dificuldades econômicas. O grupo com renda inferior às despesas apresentou maior pontuação média para a subescala “qualidade técnica”, o que reflete a satisfação principalmente dos profissionais de saúde, em comparação com o grupo de maior renda. Em estudo com mulheres desfavorecidas, as barreiras de acesso aos serviços de saúde incluíram questões como a mudança de pessoal de saúde a cada vez, seus comportamentos desrespeitosos, não considerar as necessidades dos pacientes, não dedicar tempo suficiente aos CP e ser imprudente^(21)^. Por outro lado, aspectos satisfatórios dos serviços de saúde incluíram hospitais bem equipados (56,1%), atitude positiva de médicos e profissionais de saúde (55,1%) e competência de médicos e profissionais de saúde (46,2%)^(22)^.

Em comparação com a literatura, os resultados do presente estudo sugerem que a prestação de serviços de saúde regulares e gratuitos em hospitais públicos para migrantes que adquiriram cartões de identificação de cidadania ou proteção temporária na Turquia^(23,24)^ é satisfatória para aqueles com baixa renda, mas insuficiente para atender às necessidades daqueles com maior renda (por exemplo, filas de espera, períodos de espera, ambiente físico etc.).

Questões de transporte são outro fator significativo que restringe o acesso a serviços de PC para mulheres grávidas sírias. A taxa de menos de quatro visitas ao CP é de 89,7% e a pontuação da subescala de acessibilidade foi menor entre esses indivíduos, o que indica claramente barreiras de transporte. No estudo de Penman et al.^(21)^, a distância entre a casa e o hospital, bem como a disponibilidade ou solidez do transporte público, foram identificadas como barreiras para o acesso aos serviços de saúde. No presente estudo, 45,9% dos participantes residiam em áreas rurais, o que pode ter impedido a continuidade do atendimento integral no hospital localizado na cidade. Outro achado do estudo é que 78,8% dos participantes não possuíam previdência social, o que também pode ter contribuído para esse desfecho. A literatura também sugere que ter seguridade social não só facilita o acesso aos serviços de saúde, mas também aumenta a satisfação com esses serviços^(10,25)^. Como 80,8% das gestantes sírias deste estudo ainda não obtiveram a cidadania, podem não estar informadas sobre o acesso gratuito aos serviços de saúde com carteira de identidade temporária.

A presença de crianças menores de 5 anos (73,97%) também pode afetar indiretamente o número de consultas de acompanhamento. Aquelas com um filho menor de cinco anos apresentaram maiores escores de satisfação, qualidade técnica e adequação ao CP. No estudo de Penman et al.^(21)^, todas as mulheres mencionaram questões sociais (falta de apoio social da família ou amigos, responsabilidades familiares etc.) como barreiras para acessar o CP ou receber cuidados. Em relação às responsabilidades familiares e aos afazeres domésticos, as mulheres afirmaram que cuidar de outras crianças às vezes as impede de comparecer às consultas e, quando levam seus filhos às consultas de CP, não são bem recebidas pelos profissionais de saúde. Em um estudo em que as mulheres mencionaram que o número de filhos faz parte de sua cultura, elas afirmaram receber reações negativas por isso, o que acharam perturbador^(26)^. Os resultados do estudo são consistentes com a literatura. Ter mais filhos como parte da cultura e a falta de apoio social suficiente podem afetar a satisfação mulheres sírias grávidas com o atendimento hospitalar, especialmente quando expostas a reações dos profissionais de saúde ao se apresentarem no hospital com seus filhos.

Afirmaram receber 80,1% dos CP de enfermeiras ou parteiras. Houve diferença significativa nos escores médios das subescalas de qualidade técnica, ambiente físico e adequação entre aqueles que receberam CP de enfermeiras ou parteiras e aquelas que receberam de médicos. Aqueles que receberam cuidados de enfermeiras ou parteiras obtiveram escores mais elevados. Estudo realizado na Itália constatou que as gestantes confiavam mais nas parteiras, com porcentagem de 81,2%^(27)^. Resultado semelhante sugere que a empatia e o cuidado espiritual das parteiras tiveram um efeito positivo na satisfação das mulheres (qualidade técnica e adequação). Em estudo realizado na Noruega, no entanto, não houve diferença significativa em termos de insatisfação entre as mulheres que receberam assistência ao parto de profissionais parteiras ou médicos obstetras^(10)^. A avaliação das gestantes sírias no presente estudo pode ser explicada pelas diferenças nas estruturas culturais.

Neste estudo, 70,5% das participantes afirmaram que suas gestações foram planejadas. Aquelas com gestações planejadas apresentaram escores mais altos nas subescalas arte de cuidar e acessibilidade. A literatura também enfatiza que a gravidez planejada tem um efeito positivo na satisfação com os serviços de saúde^(28)^. O escore de satisfação das gestantes representou mais da metade do escore total. No entanto, a satisfação derivada do serviço é possivelmente aumentada devido à importância da gravidez planejada para a família e ao fato de os cuidados de saúde serem gratuitos para os migrantes com carteira de identidade.

## LIMITAÇÕES DO ESTUDO

- Este estudo limitou-se a mulheres grávidas sírias que se inscreveram no Mardin Training and Research Hospital. Portanto, os resultados não podem ser generalizados para a população geral de refugiados sírios.
- A pesquisa foi realizada entre 15 de agosto e 16 de setembro de 2023. Estudos de longo prazo podem produzir resultados diferentes.
- Como as participantes foram selecionadas usando um método de amostragem intencional, a amostragem aleatória não foi realizada, limitando a generalização dos achados.
- Apenas mulheres grávidas sírias que falavam turco foram incluídas no estudo. Isso pode ter levado à exclusão daquelas que enfrentam maiores desafios no acesso aos serviços de saúde devido a barreiras linguísticas.
- A satisfação dos participantes com os serviços de saúde pode ter sido influenciada por normas culturais, que devem ser levadas em consideração.

## CONCLUSÃO

Os resultados do estudo indicam que a falta de informação, as dificuldades econômicas, a falta de seguridade social e as questões de transporte são as principais barreiras ao acesso à saúde para mulheres grávidas migrantes.

Fatores como receber CP de parteiras ou enfermeiras, ter planejado gestações e ter previdência social influenciaram a satisfação com CP das gestantes do estudo. Esses resultados lançam luz sobre o desenvolvimento de recomendações para melhorar a satisfação com os CP. Com base nestes resultados:

- As mulheres migrantes devem receber informações sobre a importância dos serviços de CP.
- Os profissionais de saúde devem receber treinamento sobre empatia e sensibilidade cultural em relação às mulheres migrantes.
- Transporte gratuito. O apoio local deve ser fornecido para reduzir os problemas relacionados ao transporte.
- O escopo da seguridade social deve ser expandido para permitir que mais mulheres migrantes se beneficiem de serviços de saúde gratuitos.
- Novos estudos em diferentes regiões com amostras maiores podem enriquecer ainda mais a base de conhecimento sobre o assunto.

# Translation: Evaluación de la satisfacción de las embarazadas sirias con la atención prenatal: estudio transversal*

**DOI:** 10.1590/1980-220X-REEUSP-2025-0002es

**Authors:** Sibel İçke, Sema Çifçi

**Affiliations:** 1 Mardin Artuklu University, Faculty of Health Sciences, Midwifery Department, Artuklu, Mardin, Türkiye.; 2 Mardin Artuklu University, Faculty of Health Sciences, Nursing Department, Artuklu Mardin, Türkiye.

**Keywords:** Migrantes, Personas Embarazadas, Atención Prenatal, Satisfacción Personal

## Abstract

**Objetivo:**

Este estudio tiene como objetivo examinar los niveles de satisfacción de las mujeres migrantes sirias embarazadas que viven en Mardin con los servicios de atención prenatal y los factores que influyen en su satisfacción.

**Método:**

Se trata de un estudio transversal. La población del estudio consistió en mujeres embarazadas sirias que solicitaron atención en el Hospital de Formación e Investigación de Mardin entre el 15 de agosto de 2023 y el 16 de septiembre de 2023. Participaron en el estudio 146 embarazadas sirias que cumplían los criterios de inclusión. Como instrumentos de recogida de datos se utilizaron el formulario de información sociodemográfica y la Escala de satisfacción con la atención prenatal.

**Resultados:**

La tasa de las que recibieron cuidados prenatales de una matrona/enfermera es del 80,1% y la de las que recibieron menos de 4 cuidados prenatales fue del 89,7%. La razón más común para no recibir una atención prenatal adecuada fue la falta de información, con una tasa del 39,7%. La puntuación media del PCSS fue de 73,39 ± 14,78.

**Conclusiones:**

Los resultados del estudio indican que la falta de información es una de las principales barreras para el acceso a la atención sanitaria de las mujeres embarazadas inmigrantes. Además, recibir servicios de atención prenatal por parte de matronas/enfermeras afectaba a la satisfacción con la atención prenatal.

## INTRODUCCIÓN

La atención prenatal (AP) se refiere a los servicios de salud prestados por profesionales de la salud a las mujeres embarazadas a lo largo del embarazo para monitorear y mantener la salud óptima de la madre y el feto^(1,2)^. La atención prenatal de alta calidad es crucial, ya que incorpora intervenciones que promueven la salud de la madre y el feto, identifica posibles afecciones adversas en una fase temprana y facilita las intervenciones oportunas^(2)^. Aunque las tasas mundiales de mortalidad materna disminuyeron aproximadamente un 34% entre 2000 y 2020, estas tasas siguen siendo significativamente elevadas en los países de ingresos bajos y medianos, ya que representan el 95% de todas las muertes maternas^(3)^.

La guerra civil que comenzó en Siria en 2011 provocó una recesión económica^(4,5)^ y desencadenó una migración generalizada. Turquía, sin duda, ha sido el país más afectado por la migración de sirios^(5,6)^. Para el año 2024, el número de sirios bajo protección temporal en Turquía es de aproximadamente 3,1 millones, de los cuales casi 1,5 millones son mujeres. Entre estas mujeres, aproximadamente 725,000 tienen entre 15 y 49 años^(7)^.

Uno de los mayores problemas a los que se enfrentan los migrantes sirios cuando se trasladan a otros países son las cuestiones relacionadas con la salud^(8)^. La falta de acceso a la AP es uno de estos retos. Ser inmigrante es uno de los factores que influyen en el acceso a la AP. Las mujeres migrantes se benefician con menos frecuencia de los servicios de AP debido a barreras como el idioma, la educación y el transporte^(9,10)^. Según los datos de la Encuesta Demográfica y de Salud de Turquía de 2018, el 11% de las migrantes sirias no reciben ninguna AP, y el 33,8% reciben una atención insuficiente, siendo significativamente menor en comparación con las mujeres embarazadas nativas^(11)^. En un estudio realizado con mujeres refugiadas sirias, la tasa de las que no recibieron AP fue del 41,3%, mientras que fue del 7,7% entre las ciudadanas turcas^(12)^. La Organización Mundial de la Salud (OMS) recomienda recibir atención prenatal (AP) al menos ocho veces durante el embarazo. Sin embargo, la cobertura mundial de la atención prenatal sigue siendo insuficiente. Según los datos de la Organización Mundial de la Salud y las Estadísticas Sanitarias Mundiales de 2016, solo el 64% de las mujeres de todo el mundo recibieron AP cuatro o más veces durante sus embarazos^(13)^.

La satisfacción es crucial en todos los ámbitos de la atención sanitaria, incluida la AP^(9)^. Los estudios revelan que las mujeres migrantes manifiestan una menor satisfacción con los servicios de salud debido al retraso de la AP, la información insuficiente y los problemas de comunicación^(14–16)^. La insatisfacción con los servicios de AP puede provocar dificultades para adaptarse a los sistemas de salud del país de migración. El presente estudio tiene como objetivo examinar los niveles de satisfacción de las mujeres embarazadas migrantes sirias que viven en Mardin con los servicios de AP y los factores que influyen en su satisfacción.

Preguntas de investigación:

1. ¿Cuáles son los niveles de satisfacción de las embarazadas sirias con los servicios de AP?
2. ¿Qué factores influyen en los niveles de satisfacción de las embarazadas sirias con los servicios de AP?

## MÉTODO

### Tipo de Investigación

Se trata de un estudio transversal.

### Población y Muestra de la Investigación

La población del estudio estuvo formada por mujeres embarazadas sirias que acudieron al Hospital de Formación e Investigación de Mardin entre el 15 de agosto de 2023 y el 16 de septiembre de 2023. Dado que el estudio se realizó exclusivamente con mujeres embarazadas migrantes sirias ingresadas en el hospital, las participantes se seleccionaron mediante el método de muestreo intencional. Un total de 146 embarazadas sirias que cumplían los criterios de inclusión participaron en el estudio. Para evaluar la potencia estadística del estudio, se realizó un análisis de potencia post-hoc para la prueba t de una muestra utilizando el software G*Power 3,1. En el análisis se utilizaron un tamaño del efecto moderado (d = 0,5), un nivel de significación estadística (α = 0,05) y el tamaño total de la muestra (n = 146). La potencia obtenida se calculó como (1-β) = 0,9999. Este resultado muestra que el estudio tiene una potencia estadística bastante alta para detectar un tamaño del efecto moderado.

### Criterios de Inclusión

1. Ser siria,
2. Tener un periodo gestacional entre 36 y 40 semanas,
3. No tener barreras lingüísticas.

### Criterios de Exclusión

1. No ser siria,
2. Tener un periodo gestacional fuera del rango de 36–40 semanas,
3. Tener barreras lingüísticas.

### Herramientas Para Recolectar Datos

En el estudio se utilizó un cuestionario que constaba de dos secciones. La primera sección recogía información sociodemográfica, mientras que la segunda incluía la Escala de Satisfacción con los Cuidados Prenatales (Prenatal Care Satisfaction Scale ***-*** PCSS), cuya validez y fiabilidad en turco fueron evaluadas por Aslantekin Özçoban et al.^(17)^ y publicadas en 2020.

- ***Formulario de identificación individual (FII):*** Este formulario, desarrollado por los investigadores basándose en la bibliografía, incluía información sociodemográfica y obstétrica y constaba de 36 preguntas.

- ***Escala de satisfacción con los cuidados prenatales (Prenatal Care Satisfaction Scale - PCSS):*** La escala fue desarrollada originalmente por Raube et al.^(18)^ en 1998 para medir la satisfacción de las mujeres embarazadas con la AP^(17,18)^. Aslantekin Özçoban et al.^(17)^ la adaptaron al turco y validaron su fiabilidad en 2020. Se trata de una escala autoinformada tipo Likert que consta de 22 ítems. Basada en la literatura, evalúa cinco subescalas: el arte de la atención, la calidad técnica, el entorno físico, la accesibilidad y la idoneidad.

*Subescala de arte de la atención:* Esta subescala incluye el valor percibido de los servicios recibidos en relación con el coste/premio pagado, la capacidad del personal para proporcionar confort y sensación de seguridad, la atención de médicos, parteras y enfermeras, el comportamiento respetuoso de los profesionales de salud y la empatía y comprensión mostradas por el personal de recepción durante estados emocionales como ansiedad, excitación o enfado. También evalúa la actitud respetuosa del personal de recepción y el interés y el enfoque mostrados por el personal de acogida/registro.

*Subescala de calidad técnica:* Incluye la adecuación de las explicaciones dadas por las parteras, enfermeras o médicos sobre los procedimientos, la competencia de los médicos, parteras y enfermeras en la prestación de los cuidados y la gestión del parto, la atención de los reconocimientos médicos y la adecuación de los equipos y suministros médicos.

*Subescala de accesibilidad:* Esta subescala evalúa las condiciones de transporte, el tiempo de espera para el examen en una cita, la duración entre la fecha de la cita inicial y la fecha prevista para el siguiente seguimiento, y la idoneidad de los horarios de apertura (temprano o tarde).

*Subescala de medio físico:* Evalúa la limpieza, comodidad y conveniencia de las instalaciones, el estado de las zonas de exploración y la adecuación de las salas de espera.

*Subescala de Adecuación:* Incluye el tiempo dedicado por el personal a proporcionar asesoramiento nutricional durante el embarazo, la competencia profesional de médicos, parteras y enfermeras, y la viabilidad del asesoramiento proporcionado por los profesionales de salud a lo largo del embarazo para proteger la salud tanto de la madre como del lactante.

Los valores alfa de Cronbach para las subescalas oscilan entre 0,7 y 0,9, mientras que es de 0,95 para la escala global. No hay punto de corte en la escala. La puntuación más baja fue de 22, mientras que la más alta fue de 110. Las respuestas de la escala se califican del siguiente modo: deficiente (1), regular (2), bien (3), muy bien (4) y excelente (5). A medida que aumenta la puntuación en la escala, la satisfacción se considera alta, y a medida que disminuye la puntuación, la satisfacción se considera baja (18).

### Consideraciones Éticas

Antes de realizar el estudio, se obtuvo la aprobación del Comité Ético de Investigación Clínica No Intervencionista de la Universidad Mardin Artuklu, con el número de decisión 2022-9, de fecha 01/08/2022. La Coordinación de Proyectos de Investigación Científica de la Universidad Mardin Artuklu proporcionó apoyo financiero para el estudio a través del proyecto MAÜ.BAP,22.SBF,026, destinado a llevar a cabo la investigación.

### Recolección de Datos

Los datos del estudio se recolectaron mediante un método de encuesta cara a cara en las consultas externas de obstetricia y ginecología, la clínica de NST (prueba de no estrés) y las clases prenatales. Los cuestionarios se realizaron en turco cara a cara con las embarazadas que aceptaron participar en el estudio. Antes de iniciar el estudio, se explicaron su finalidad y metodología, y se informó a las participantes de que la participación era voluntaria y de que podían retirarse del estudio en cualquier momento. Se obtuvo el consentimiento escrito y verbal de las participantes. Para eliminar la barrera lingüística, la comunicación con los participantes se estableció en turco y se excluyó del estudio a aquellos que se enfrentaban a una barrera lingüística. Los cuestionarios se rellenaron en unos 10 minutos.

### Análisis de los Datos

Los datos se analizaron con el paquete estadístico SPSS (Statistical Package for Social Sciences) versión 20. Se calcularon las estadísticas descriptivas de los participantes, incluidas las frecuencias, los porcentajes y las medias. Para todos los análisis, el umbral de significación estadística se fijó en p < 0,05. Se calculó la puntuación media total de las escalas, utilizando la prueba de normalidad de Shapiro-Wilk para determinar la distribución normal de las puntuaciones de las escalas. Dado que las puntuaciones de las escalas tenían una distribución normal, se realizó una prueba ANOVA unidireccional para evaluar la significación de las diferencias entre las medias de tres o más grupos y se utilizó la prueba de Bonferroni como prueba post hoc para identificar qué grupos contribuían a las diferencias significativas. Se utilizó la prueba t independiente para comparar las puntuaciones medias de dos grupos independientes.

## RESULTADOS

La Tabla 1 muestra las características sociodemográficas de las embarazadas.

**Tabla 1:** Características sociodemográficas de las mujeres embarazadas. Mardin, Turquía, 2023.

Características		n	%
Edad (años)	16–20	20	13,7
	**21–25**	**44**	**30,1**
	26**–**30	43	29,5
	31–35	25	17,1
	36 and ↑	14	9,6
Proceso de ciudadanía	Si	28	19,2
	**Todavía no**	**118**	**80,8**
Lugar de residencia	Provincia	44	30,1
	**Distrito**	**67**	**45,9**
	Pueblo	35	24,0
Tipo de familia	Familia extendida	46	31,5
	**Familia nuclear**	**100**	**68,5**
Nivel educativo	Analfabeta	42	28,8
	**Primaria completa**	**62**	**42,5**
	Secundaria completa	31	21,2
	Estudios superiores y más	11	7,5
Nivel educativo del esposo	Analfabeto	32	21,9
	**Primaria completa**	**67**	**45,9**
	Secundaria completa	34	23,3
	Estudios superiores y más	13	8,9
Estatus de empleo	Empleada	12	8,2
	**Desempleada**	**134**	**91,8**
Estatus de empleo del esposo	Empleado	128	87,7
	**Desempleado**	18	12,3
Nivel de ingresos	**Ingresos menores que gastos**	**85**	**58,2**
	Ingresos iguales a gastos	58	39,7
	Ingresos mayores que gastos	3	2,1
Tipo de vivienda	Apartamento	51	34,9
	**Casa**	**95**	**65,1**
Seguridad social	Tiene	31	21,2
	No tiene	**115**	**78,8**
Tiempo residencia en Turquía (años)	Menos de 5	12	8,2
	**6–10**	**95**	**65,1**
	> 10	39	26,7
Número de personas en el hogar	**2–5**	**86**	**58,9**
	6–10	55	37,7
	11 y más	5	3,4
*Número de niños menores de 5 años (N = 108)	1	46	42,6
	**2**	**47**	**43,5**
	3 y más	15	13,9
Presencia de enfermedades crónicas	Si	18	12,3
	**No**	**128**	**87,7**
*Enfermedad crónica	Asma	1	5,5
	Diabetes	2	11,2
	**Bocio**	**7**	**38,9**
	Renal	1	5,5
	Hipertensión	7	38,9
Total		**146**	**100**

*Los porcentajes se calcularon a partir de las respuestas dadas.

Según la tabla, su edad media es de 27,2 ± 5,89, con un 30,1% en el grupo de 21–25 años. De ellas, el 80,8% aún no ha obtenido la nacionalidad, el 45,9% vive en el distrito y el 68,5% tiene una familia nuclear. En cuanto al nivel de estudios, el 42,5% de las embarazadas y el 45,9% de sus maridos tienen estudios primarios. De las embarazadas, el 91,8% no tiene empleo, mientras que esta tasa es del 12,3% para sus maridos, el 58,2% tiene ingresos inferiores a sus gastos, el 65,1% vive en una vivienda unifamiliar y el 78,8% no tiene seguridad social. Entre ellas, el 65,1% lleva viviendo en Turquía entre 6 y 10 años. De ellos, el 58,9% declara que el número de personas en su hogar oscila entre 2 y 5, el 43,5% tiene dos hijos menores de 5 años y el 87,7% declara no padecer ninguna enfermedad crónica. Entre los que padecen enfermedades crónicas (12,3%), el 38,9% tiene bocio y el mismo porcentaje (38,9%) sufre hipertensión.

La Tabla 2 presenta la fecundidad y el estado civil de las mujeres embarazadas.

**Tabla 2:** Fecundidad y estado civil de las mujeres mbarazadas Fecundidad y estado civil de las mujeres embarazadas – Mardin, Turquía, 2023.

Características		n	%
Semanas de gestación	36	27	18,5
	37	34	23,3
	**38**	**44**	**30,1**
	39	30	20,5
	40	11	7,5
Atenciones prenatales (AP) recibida de	**Partera/enfermera**	**117**	**80,1**
	Médico	29	19,9
Número de AP recibidas	**< 4**	**131**	**89,7**
	≥ 4	15	10,3
*Razón para recibir menos de 4 AP (N = 131)	Familiares	4	3,1
	**Ignorancia**	**52**	**39,7**
	Falta de dinero	35	26,7
	Problemas de salud	3	2,3
	Transporte	23	17,5
	Falta de tiempo	14	10,7
Número de embarazos	1	25	17,1
	**2 y más**	**121**	**82,9**
Edad al matrimonio (años)	13–18	58	39,7
	**19**–**25**	**79**	**54,1**
	26 y más	9	6,2
Tiempo de casada (años)	**1**–**10**	**108**	**74,0**
	11–25	38	26,0
Edad al primer embarazo (años)	**13**–**18**	**46**	**31,5**
	19-25	81	55,5
	26 y más	19	13,0
Separación entre embarazos (N = 119) (años)	1	32	26,9
	**2**	**36**	**30,2**
	3	19	16,0
	4 y más	32	26,9
*Número de hijos (N = 119)	1	28	23,5
	2	30	25,2
	**3**	**31**	**26,1**
	4 y más	30	25,2
Tuvo problemas de salud durante el embarazo (N = 146)	**Si**	**24**	**16,4**
	No	122	83,6
*Tipo de problemas (N = 24)	Sangrado	4	16,6
	Edema	4	16,6
	**Diabetes**	**6**	**25,0**
	Vómitos	1	4,2
	Hipertensión	4	16,6
	Hipotensión	1	4,2
	Infección	1	4,2
	Retardo en el desarrollo	1	4,2
	Riesgo de parto prematuro	2	8,4
Embarazo planificado	**Si**	**103**	**70,5**
	No	43	29,5
Vía de nacimiento esperada	**Vaginal**	**93**	**63,7**
	Cesárea	53	36,3
Vía de nacimiento preferida	**Vaginal**	**101**	**69,2**
	Cesárea	45	30,8
Sexo fetal	**femenino**	**74**	**50,7**
	masculino	55	37,7
	Desconocido	17	11,6
Total		165	100,0

*Los porcentajes se calcularon a partir de las respuestas dadas.

Así, el 30,1% de las embarazadas se encontraba en la semana 38 de gestación, el 82,9% había tenido dos o más embarazos, el 54,1% se casó entre los 19 y los 25 años, el 31,5% tuvo su primer embarazo entre los 13 y los 18 años, y el 30,2% tenía una duración de 2 años entre su embarazo anterior y el actual. De ellas, el 26,1% tenía tres hijos, el 16,4% declaró haber tenido problemas de salud durante este embarazo, y entre las que tenían problemas de salud, el 25,0% declaró que el problema de salud era la diabetes.

La distribución estadística descriptiva y los valores del Alfa de Cronbach del PCSS y sus subescalas se presentan en la Tabla 3.

**Tabla 3:** Distribución estadística descriptiva y valores alfa de Cronbach de la PCSS y sus subescalas – Mardin, Turquía, 2023.

Escala y subescalas	N	Min.	Max.	Media	DE	Alfa de Cronbach (α)
*Escala de satisfacción con los cuidados prenatales (PCSS)*	146	35,00	104,00	73,39	14,78	0,93
El arte del cuidado	146	7,00	25,00	16,69	3,75	0,81
Calidad técnica	146	7,00	24,00	17,27	3,70	0,75
Accesibilidad	146	3,00	15,00	9,11	2,66	0,71
Medio físico	146	5,00	20,00	13,06	3,21	0,72
Adecuación	146	4,00	15,00	10,27	2,25	0,73

DE = Desvío estándar.

La puntuación media del PCSS es de 73,39 ± 14,78. En el presente estudio, el coeficiente alfa de Cronbach fue de 0,93. La comparación de las puntuaciones totales y de las subescalas del PCSS con algunas características de las embarazadas se presenta en la Tabla 4.

**Tabla 4:** Comparación de las puntuaciones totales y de las subescalas del PCSS con algunas características de las mujeres embarazadas – Mardin, Turquía, 2023.

Características		PCSS	El arte del cuidado	Calidad técnica	Accesibilidad	Medio físico	Adecuación
Lugar de vivienda*	Pueblo^a^	69,71 ± 15,81	16,34 ± 3,84	16,22 ± 4,04	7,71 ± 2,85	12,42 ± 3,44	10,02 ± 2,18
	Distrito^b^	75,95 ± 14,31	17,19 ± 3,61	17,88 ± 3,55	9,59 ± 2,41	13,77 ± 3,12	10,47 ± 2,23
	Provincia^c^	72,43 ± 14,20	16,22 ± 3,88	17,18 ± 3,52	9,50 ± 2,51	12,50 ± 2,99	10,15 ± 2,33
	**Análisis estadístico**	F = 2,221 P = 0,11	F =1,087 P = 0,34	F =1,087 P = 0,34	**F = 6,931** **P = 0,00**	**F = 3,098** **P = 0,04**	F = 0,539 P = 0,58
	**Diferencia**				a-b-c. a-b. a–c		
Sexo fetal*	Femenino^a^	75,87 ± 13,26	17,24 ± 3,34	17,95 ± 3,25	9,44 ± 2,53	13,50 ± 3,057	10,64 ± 2,14
	Masculino^b^	72,32 ± 14,56	16,65 ± 3,79	16,80 ± 3,64	9,07 ± 2,54	12,87 ± 3,055	9,90 ± 2,25
	Desconocido^c^	66,05 ± 19,25	14,47 ± 4,66	15,82 ± 5,08	7,82 ± 3,26	11,82 ± 4,08	9,82 ± 2,50
	**Análisis estadístico**	**F = 3,39** **P = 0,03**	**F =3,923** **P = 0,02**	F = 3,113 **P = 0.04**	F =2,639 P = 0,07	F = 2,078 P = 0,12	F = 2,131 P = 0,12
	**Diferencia**	a–c	a–c	a–c			
Nivel de ingresos*	Ingresos menos que gastos	74,87 ± 14,49	16,84 ± 3,74	17,90 ± 3,58	9,25 ± 2,55	13,30 ± 3,22	10,49 ± 2,18
	Ingresos iguales que gastos	71,79 ± 14,91	16,65 ± 3,69	16,55 ± 3,67	8,94 ± 2,81	12,79 ± 3,23	9,94 ± 2,35
	Ingresos mayores que gastos	62,66 ± 18,03	13,33 ± 5,03	13,33 ± 3,78	8,33 ± 3,51	11,66 ± 2,08	10,33 ± 1,15
	**Análisis estadístico**	F = 1,56 P = 0,21	F =1,279 P = 0,28	F = 4,222 **P = 0,01**	F = 0,364 P = 0,69	F = 0,729 P = 0,48	F = 1,020 P = 0,36
	**Diferencia**			a–c			
Tiempo en Turquía años*	Menos de 5^a^	76,16 ± 12,91	17,66 ± 3,55	17,75 ± 3,048	8,41 ± 3,28	14,08 ± 2,93	10,75 ± 2,37
	6–10^b^	71,78 ± 15,96	16,43 ± 4,00	16,93 ± 3,92	8,80 ± 2,76	12,78 ± 3,50	10,03 ± 2,33
	Más de 10^c^	76,46 ± 11,66	17,05 ± 3,13	17,94 ± 3,26	10,10 ± 1,87	13,43 ± 2,42	10,71 ± 1,93
	**Análisis estadístico**	F = 1,625 P = 0,20	F = 30,808 P = 0,44	F = 1,143 P = 0,32	**F = 3,915** **P = 0,02**	F = 1,217 P = 0,29	F = 1,599 P = 0,20
	**Diferencia**				b-c		
Número de niños menores de cinco años*	1^a^	75,54 ± 14,97	17,30 ± 3,98	17,95 ± 3,74	9,26 ± 2,74	13,23 ± 3,42	10,69 ± 2,22
	2^b^	73,50 ± 14,91	16,39 ± 3,80	17,10 ± 3,74	9,21 ± 2,39	13,28 ± 3,38	10,32 ± 2,10
	3 y más^c^	62,93 ± 15,18	14,53 ± 3,83	14,60 ± 4,10	7,66 ± 2,60	11,20 ± 2,95	8,86 ± 2,41
	**Análisis estadístico**	**F = 4,073** **P = 0,20**	F = 2,915 P = 0,06	**F = 4,424** **P = 0,01**	F = 2,400 P = 0,09	F = 2,449 P = 0,09	**F = 3,909** **P = 0,02**
	**Diferencia**	a–c		a–c			a–c
De quien recibió la AP**	Partera/enfermera	74,52 ± 14,66	16,81 ± 3,80	17,59 ± 3,70	9,20 ± 2,57	13,39 ± 3,13	10,46 ± 2,09
	Médico	68,86 ± 14,63	16,24 ± 3,62	16,00 ± 3,48	8,76 ± 3,02	11,76 ± 3,26	9,52 ± 2,71
	**Análisis estadístico**	t = 1,862 p = 0,06	t = 0,731 p = 0,46	**t = 2,094** **p = 0,04**	t = 0,808 p = 0,42	**t = 2,498** **p = 0,01**	**t = 2,050** **p = 0,04**
Número de AP**	< 4	72,62 ± 14,92	16,56 ± 3,78	17,11 ± 3,73	8,86 ± 2,60	12,98 ± 3,30	10,19 ± 2,23
	≥ 4	80,20 ± 11,78	17,87 ± 3,40	18,67 ± 3,27	11,40 ± 2,13	13,80 ± 2,27	11,00 ± 2,30
	**Análisis estadístico**	t = -1,899 p = 0,06	t = -1,274 p = 0,20	t = –1,546 p = 0,12	**t =** –**3,655** **p = 0,00**	t = –1,246 p = 0,23	t = –1,326 p = 1,19
Embarazo planificado**	Si	74,82 ± 14,45	17,18 ± 3,56	17,38 ± 3,67	9,43 ± 2,55	13,31 ± 3,17	10,38 ± 2,32
	No	69,97 ± 15,14	15,53 ± 3,99	17,00 ± 3,80	8,34 ± 2,78	12,48 ± 3,25	10,00 ± 2,03
	**Análisis estadístico**	t = 1,821 p = 0,07	**t = 0,479** **p = 0,01**	t = –1,695 p = 0,092	**t =** –**2,647** **p = 0,009**	t = 1,415 p = 0,159	t = 0,952 p = 0,343
Seguridad Social**	Hiene	77,48 ± 11,23	17,54 ± 3,09	17,90 ± 2,80	9,80 ± 2,28	13,93 ± 2,64	10,74 ± 1,86
	No tiene	72,29 ± 15,45	16,46 ± 3,89	17,10 ± 3,90	8,93 ± 2,73	12,83 ± 3,31	10,14 ± 2,32
	**Análisis estadístico**	**t = 2,092** **p = 0,04**	t = 1,424 p = 0,15	t = 0,576 p = 0,56	**t = 0,468** **p = 0,02**	t = 1,415 p = 0,15	t = 0,952 p = 0,34

*Prueba Anova de una vía;

**Prueba T de muestras independientes.

Según la tabla, no hay diferencias significativas en sus puntuaciones de edad, educación, educación del cónyuge, número de personas en el hogar, semana gestacional, edad de matrimonio, edad en el primer embarazo, año de matrimonio, duración desde el embarazo anterior, número de hijos, situación de ciudadanía, situación laboral de las mujeres y sus cónyuges, tipo de residencia, tipo de familia, enfermedades crónicas, problemas relacionados con el embarazo y método de parto esperado y preferido (p > 0,05). Sin embargo, existe una correlación significativa entre el lugar de residencia y el entorno físico y la accesibilidad (p < 0,05). La correlación es significativa entre el sexo del feto y la puntuación total de la PCSS, así como las subescalas de calidad asistencial y calidad técnica (p < 0,05). Existe una diferencia significativa entre la duración de la residencia en Turquía y la subescala de accesibilidad (p < 0,05). Existe una diferencia significativa entre el número de hijos menores de cinco años y la puntuación total de la PCSS, así como las subescalas de calidad técnica y accesibilidad (p < 0,05). Existe una diferencia significativa entre el nivel de ingresos y las subescalas de calidad técnica (p < 0,05).

## DISCUSIÓN

Las refugiadas embarazadas y las mujeres desfavorecidas se consideran un grupo vulnerable. Este grupo tiene un mayor riesgo de sufrir problemas graves de salud neonatal y materna debido a factores como un mayor riesgo de cesárea, unas condiciones de salud primaria inadecuadas y unos procesos de embarazo más complejos^(19)^.

En el estudio, las razones más comunes por las que las embarazadas sirias no reciben suficiente AP son «desconocimiento» (39,7%), «dificultades económicas» (26,7%) y «problemas de transporte» (17,5%). El hallazgo relacionado con la ignorancia coincide con la literatura que sugiere que la falta de conocimiento sobre los servicios de AP impide la asistencia regular a las revisiones^(20)^. En particular, la falta de concienciación sobre la importancia de la asistencia sanitaria es significativa para los grupos de inmigrantes. Esto puede impedir que las mujeres inmigrantes gestionen su embarazo de forma más consciente y utilicen los servicios de salud con regularidad. Además, estudios anteriores indican que las mujeres inmigrantes suelen estar menos satisfechas con los servicios de atención prenatal debido a factores como el retraso en el acceso, la información insuficiente y los problemas de comunicación con los profesionales de salud^(10)^. De forma similar a nuestros hallazgos, estas barreras a menudo dan lugar a una atención prenatal inconsistente o inadecuada.

La segunda razón más común es la «dificultad económica», que puede estar relacionada con el hecho de que la mayoría de las participantes (58,2%) tenían unos ingresos inferiores a sus gastos. Los resultados de nuestro estudio ponen de manifiesto las dificultades a las que se enfrenta el acceso a los servicios de salud, sobre todo debido a las dificultades económicas. El grupo con ingresos inferiores a los gastos presenta una puntuación media más alta en la subescala «calidad técnica», que refleja la satisfacción debida principalmente al personal sanitario, en comparación con el grupo con ingresos más elevados. En un estudio realizado con mujeres desfavorecidas, las barreras para acceder a los servicios de salud incluían cuestiones como el cambio de personal sanitario cada vez, sus comportamientos irrespetuosos, no tener en cuenta las necesidades de los pacientes, no dedicar tiempo suficiente a la AP y ser desconsiderados^(21)^. En otro estudio, por el contrario, los aspectos satisfactorios de los servicios de salud incluían hospitales bien equipados (56,1%), la actitud positiva de los médicos y el personal sanitario (55,1%) y la competencia de los médicos y el personal sanitario (46,2%)^(22)^. En comparación con la bibliografía, los resultados del presente estudio sugieren que la prestación de servicios de salud estándar regulares y gratuitos en los hospitales públicos para los inmigrantes que han adquirido la nacionalidad o tarjetas de identificación de protección temporal en Turquía^(23,24)^ es satisfactoria para las personas con ingresos bajos, pero insuficiente para satisfacer las necesidades de las personas con ingresos más elevados (por ejemplo, colas, periodos de espera, entorno físico, etc.).

Los problemas de transporte son otro factor importante que restringe el acceso de las embarazadas sirias a los servicios de AP. La tasa de las que reciben menos de cuatro visitas de AP es del 89,7% y la puntuación de la subescala de accesibilidad es más baja entre estas personas, lo que indica claramente la existencia de barreras de transporte. En el estudio de Penman et al.^(21)^, la distancia entre el domicilio y el hospital, así como la disponibilidad o solidez del transporte público, se identificaron como barreras para acceder a los servicios de salud. En nuestro estudio, el 45,9% de los participantes vivían en zonas rurales, lo que puede haberles impedido continuar la atención completa en el hospital situado en la ciudad. Otro hallazgo del estudio es que el 78,8% de los participantes no tenían seguridad social, lo que también puede haber contribuido a este resultado. La literatura también sugiere que tener seguridad social no sólo facilita el acceso a los servicios de salud, sino que también aumenta la satisfacción con estos servicios^(10,25)^. En el presente estudio, dado que el 80,8% de las embarazadas sirias aún no han obtenido la nacionalidad, es posible que no estén bien informadas sobre el acceso gratuito a los servicios de salud con un documento de identidad temporal.

La presencia de niños menores de 5 años (73,97%) también puede afectar indirectamente al número de visitas de seguimiento. En nuestro estudio, los que tenían un hijo menor de 5 años obtuvieron puntuaciones más altas en satisfacción con el PC, calidad técnica e idoneidad. En el estudio de Penman et al.^(21)^, todas las mujeres mencionaron cuestiones sociales (falta de apoyo social por parte de familiares o amigos, responsabilidades familiares, etc.) como barreras para acceder a la AP o recibir atención. En cuanto a las responsabilidades familiares y las tareas domésticas, las mujeres afirmaron que el cuidado de otros hijos a veces les impide acudir a las citas, y cuando llevan a sus hijos a las citas de AP, no son bien recibidas por el personal sanitario. En un estudio en el que las mujeres mencionaron que el número de hijos forma parte de su cultura, afirmaron recibir reacciones negativas por ello y lo consideraron molesto^(26)^. Los resultados del estudio concuerdan con la bibliografía. Tener más hijos, como parte de su cultura, y la falta de apoyo social suficiente pueden haber afectado a la satisfacción con la atención hospitalaria de las embarazadas sirias, especialmente cuando se exponen a reacciones del personal sanitario al presentarse en el hospital con sus hijos.

Declararon que recibían el 80,1% de la AP de las parteras/enfermeras. Existe una diferencia significativa en las puntuaciones medias de las subescalas de calidad técnica, entorno físico e idoneidad entre las que recibieron AP de parteras/enfermeras y las que la recibieron de médicos. Las que recibieron atención de parteras/enfermeras obtuvieron puntuaciones más altas. En un estudio realizado en Italia, las embarazadas indicaron confiar más en las parteras, con una tasa del 81,2%^(27)^. La presencia de un resultado similar sugiere que la empatía y la atención espiritual de las parteras tuvieron un efecto positivo en la satisfacción de las mujeres (calidad técnica e idoneidad). En un estudio realizado en Noruega, sin embargo, no hubo diferencias significativas en términos de insatisfacción entre las mujeres que recibían atención en el parto por parte de practicantes, parteras u obstetras^(10)^. La evaluación de las embarazadas sirias en el presente estudio puede explicarse por las diferencias en las estructuras culturales.

En nuestro estudio, el 70,5% de las participantes declararon que sus embarazos eran planificados. Las que tenían embarazos planificados obtuvieron puntuaciones más altas en las subescalas de arte de la atención y accesibilidad. La bibliografía también destaca que los embarazos planificados tienen un efecto positivo en la satisfacción con los servicios de salud^(28)^. En el presente estudio, la puntuación de satisfacción de las embarazadas representó más de la mitad de la puntuación total. Sin embargo, es posible que la satisfacción derivada del servicio aumente debido a la importancia de los embarazos planificados para la familia y a que la asistencia sanitaria es gratuita para los inmigrantes con tarjeta de identificación.

## LIMITACIONES DEL ESTUDIO

- Este estudio se limita a las embarazadas sirias que acudieron al Hospital de Formación e Investigación de Mardin. Por lo tanto, los resultados no pueden generalizarse al conjunto de la población refugiada siria.
- La investigación se llevó a cabo entre el 15 de agosto y el 16 de septiembre de 2023. Los estudios a más largo plazo pueden arrojar resultados diferentes.
- Dado que las participantes se seleccionaron mediante un método de muestreo intencional, no se realizó un muestreo aleatorio, lo que limita la generalizabilidad de los resultados.
- Sólo se incluyeron en el estudio las embarazadas sirias que hablaban turco. Esto puede haber llevado a la exclusión de mujeres que se enfrentan a mayores dificultades para acceder a los servicios de salud debido a las barreras lingüísticas.
- La satisfacción de las participantes con los servicios de salud puede haberse visto influida por las normas culturales, que deben tenerse en cuenta.

## CONCLUSIÓN

Los resultados del estudio indican que la falta de información, las dificultades económicas, la falta de seguridad social y los problemas de transporte son las principales barreras para el acceso a la atención sanitaria de las embarazadas inmigrantes.

Factores como recibir AP de comadronas o enfermeras, tener embarazos planificados y disponer de seguridad social influyeron en la satisfacción con la AP de las embarazadas del estudio. Estos resultados arrojan luz sobre el desarrollo de recomendaciones para mejorar la satisfacción con la AP. Basándonos en estos resultados,

- Las mujeres inmigrantes deberían recibir información sobre la importancia de los servicios de AP.
- El personal sanitario debería recibir formación sobre empatía y sensibilidad cultural hacia las mujeres inmigrantes.
- Debería proporcionarse apoyo local de transporte gratuito para reducir los problemas relacionados con el transporte.
- Debería ampliarse el alcance de la seguridad social para que más mujeres inmigrantes puedan beneficiarse de los servicios de salud gratuitos.
- La realización de nuevos estudios en distintas regiones con muestras de mayor tamaño podría enriquecer aún más la base de conocimientos sobre este tema.
